# Role of MLIP in burn-induced sepsis and insights into sepsis-associated cancer progression

**DOI:** 10.3389/fimmu.2025.1540998

**Published:** 2025-02-14

**Authors:** Zhiwei Li, Qian Wang, Yezi Liu, Shuting Yang, Jin Zhao, Changdong Wu, Changmin Wang

**Affiliations:** ^1^ Clinical Laboratory Center, People’s Hospital of Xinjiang Uygur Autonomous Region, Urumqi, Xinjiang, China; ^2^ Xinjiang Emergency Center, People’s Hospital of Xinjiang Uygur Autonomous Region, Urumqi, Xinjiang, China

**Keywords:** burns, sepsis, MLIP, macrophage activation, bioinformatics, inflammation, gene expression, prognosis

## Abstract

**Introduction:**

Burn-induced sepsis is a critical clinical challenge marked by systemic inflammation, immune dysregulation, and high mortality. Macrophage-driven inflammatory pathways are central to sepsis pathogenesis, while immune cell metabolic reprogramming plays a key role in both sepsis and cancer progression.

**Methods:**

Bioinformatics analyses using GEO, TCGA, and GTEx datasets identified MLIP-modulated genes linked to immune responses and prognosis. *In vitro*, LPS-stimulated HUVEC cells were used to study MLIP’s effects on inflammation and macrophage function through cell viability, ROS levels, cytokine expression, qRT-PCR, and immunofluorescence assays.

**Results:**

MLIP-modulated genes were associated with immune-related metabolic pathways in both sepsis and cancer. Epigenetic analysis showed MLIP expression is regulated by promoter methylation and chromatin accessibility. Prognostic analyses revealed MLIP’s impact on survival outcomes across cancer types. *In vitro*, MLIP reduced inflammation, oxidative stress, and macrophage hyperactivation.

**Conclusions:**

MLIP regulates immune-metabolic dynamics in burn-induced sepsis, influencing macrophage activity and oxidative stress. Its role in metabolic reprogramming suggests MLIP as a potential therapeutic target linking immune modulation and cancer progression. Further research on MLIP’s role in immune evasion and tumor metabolism may inform novel therapeutic strategies.

## Introduction

Currently, burns, sepsis, and cancer represent significant global public health challenges, all of which involve complex immune responses characterized by inflammation, cellular injury, and subsequent repair mechanisms. A comprehensive understanding of the unique pathophysiological processes underlying these conditions is crucial for advancing therapeutic strategies. Furthermore, identifying pathways that can alleviate disease progression, rather than allowing it to worsen, holds substantial therapeutic value. Elucidating the mechanisms behind these interconnected yet distinct health issues, by synthesizing global and regional data, enables a systematic analysis of key aspects such as disease epidemiology, risk factors, and disease burden. This knowledge provides critical evidence for the development of effective control strategies and facilitates the formulation of targeted and impactful interventions (1–3).

Sepsis is notably characterized by its rapid onset and high mortality rate, primarily attributed to the excessive release of cytokine that initiates a cascade of immune imbalances leading to immunosuppression and, ultimately, multiorgan failure (4, 5). A considerable body of research has focused on the pathophysiological mechanisms through which sepsis induces organ dysfunction. With the support of immunological approaches, basic research has increasingly concentrated on the role of macrophages in various organs during this process, while also showing significant interest in the regulatory mechanisms of macrophages in the context of infection. Although macrophages play a central role, other immune cells, such as neutrophils and T cells, are equally critical in the progression of both sepsis and cancer. A thorough understanding of the immune responses of various immune cell types is vital for developing comprehensive therapeutic strategies. Understanding the role of cytokines in immune responses provides valuable insights for the development of effective treatment strategies for a range of diseases. Through research into cytokine signaling and immune regulation mechanisms, scientists have identified numerous potential therapeutic targets (6). Burn injuries induce widespread inflammatory responses and immune dysregulation, often resulting in immunosuppression and increased susceptibility to sepsis. Severe burns can lead to systemic inflammatory response syndrome (SIRS), a pathway similar to sepsis, frequently culminating in organ failure and high mortality rates (7). Investigating the immune and inflammatory alterations triggered by burns is critical for devising preventive strategies against burn-induced sepsis (8, 9).In this context, a significant number of macrophages may become hyperactivated, releasing immune mediators in a rapid, burst-like manner. This amplified immune response can shift the individual’s focus from managing the initial inflammatory phase to addressing secondary infections, which are frequently observed following severe burns and critically influence the prognosis of burn-induced sepsis. When designing therapeutic interventions, it is essential to consider the dynamic temporal patterns of macrophage activation, particularly during the acute and chronic phases of sepsis and burns, as well as their interplay with metabolic shifts. These metabolic changes can further exacerbate immune dysfunction, influencing the effectiveness of immune responses and the overall recovery process.

Additionally, the immunosuppressive state induced by burn injuries can impair the body’s capacity to detect and combat emerging cancers, potentially heightening the risk for certain malignancies (10, 11). Cellular regeneration following burn injuries may foster conditions conducive to abnormal cell growth and cancerous lesions. Such an environment could contribute to carcinogenesis (12, 13). In the pursuit of understanding the underlying mechanisms, recent research advancements in the field of molecular biology have provided valuable insights. Transcriptomics has emerged as a crucial tool, as it plays a significant role in revealing the immune microenvironment and holds great importance for the diagnosis and prognosis of various diseases (14, 15). Through transcriptome analysis and single - cell techniques, researchers are now able to identify the functional characteristics and molecular markers of different cancer - associated fibroblast (CAF) sub - populations, thereby providing a solid theoretical foundation for the development of precise treatment strategies (16). Moreover, the immune escape mechanisms prevalent in the tumor microenvironment and their subsequent promotion of tumor progression have been identified as potential therapeutic targets for novel targeted treatment approaches (17). Single - cell transcriptomics analysis has demonstrated that diverse signaling pathways and immune regulatory factors play critical roles in antigen - specific cell functional exhaustion and immune escape, offering essential guidance for optimizing treatment regimens (18, 19). By leveraging single - cell RNA sequencing and bioinformatics analysis, researchers have been successful in identifying important molecules and pathways associated with the tumor microenvironment, thus charting new directions for precision medicine (20). Additionally, single - cell transcriptomics has been applied to analyze the repair process, shedding light on the underlying mechanisms (21). Concurrently, bioinformatics analysis and experimental validation of potential biomarkers have furnished crucial indicators for disease diagnosis and prognosis assessment (22–24). The advent of novel technologies and advanced molecular research methods has had a profound impact on disease research and treatment (25). In the realm of cancer treatment, for instance, antibody - drug conjugates and photodynamic therapy have witnessed continuous development. Through meticulous mechanism research, rigorous clinical validation, and the integration of bioinformatics analysis of clinical data, these advancements have propelled cancer treatment to new heights (26). Modern bioinformatics and big data technologies are increasingly indispensable in disease diagnosis, prognosis evaluation, and treatment. The application of these cutting - edge technologies and methodologies has not only spurred the growth of biomedical research but has also laid a robust foundation for the realization of precision medicine (27, 28). Gaining insight into how burns may facilitate cancer formation could inform the development of strategies to mitigate the risk of advanced-stage cancer (13, 29). However, the specific mechanisms by which burn-induced immune-metabolic changes impact cancer development across different cancer types should be further elucidated, as this connection is not yet fully understood.

Sepsis, with its extensive infectious inflammation, similarly poses a potential pathway for cancer development (30, 31). The prolonged inflammatory state associated with sepsis may lead to genomic instability and the accumulation of mutations, creating conditions that disrupt local cellular environments and potentially transform cells into malignant tumors (32, 33). Addressing chronic sepsis-related inflammation and bolstering immune function could play a pivotal role in modulating cancer risk and progression (31, 34). Furthermore, various macrophage cell death pathways, such as pyroptosis, autophagy, and ferroptosis, play a significant role in organ damage during sepsis. These mechanisms add complexity to the immune response in sepsis and may serve as potential therapeutic targets for modulating immune responses and preventing multiorgan failure. The heterogeneity of tumor microenvironments across different cancer types may limit the generalizability of findings related to the impact of sepsis-induced immune alterations on cancer development. Moderate exercise has long been recognized for its positive effects on immune function, strengthening the body’s defense mechanisms. Such exercise may enhance management strategies for conditions like burns, sepsis, and cancer (35, 36). Exercise supports recovery from burn injuries, reduces the risk of sepsis, and mitigates some irreversible side effects of cancer therapies, while also boosting the efficacy of treatments (37, 38). Thus, this emerging perspective could foster a more integrated approach to patient care, treating these conditions in concert rather than in isolation (39, 40). Research has found that nicorandil can relieve joint contracture and fibrosis by inhibiting the RhoA/ROCK and TGF - β1/Smad signaling pathways, providing potential drug targets for joint diseases (41).

The fields of cell therapy and biologic agent therapy are developing rapidly. Studies on engineered extracellular vesicles and exosomes derived from stem cells have delved deep into their mechanisms of action and actual treatment effects in tissue repair and regeneration (42). Moreover, exploring the relationship between exercise-induced metabolites and macrophage activity offers a promising area for research. Insights from this field could reveal novel methods for immune regulation and open pathways for resolving sepsis-associated inflammation. In summary, the combined effects of conditions such as burns and sepsis may increase the risk of cancer, a factor that warrants attention both medically and socially (31, 43). Moreover, by investigating the role of macrophages and their immune functions in sepsis, we aim to identify novel therapeutic targets to reduce multiorgan dysfunction and improve patient survival rates. Moving forward, it is essential to further explore the complex interconnections between burns, sepsis, and cancer, with the goal of optimizing treatment strategies based on these findings, ultimately offering better therapeutic prospects for patients.

## Materials and methods

### Exercise-induced modulation of pan-cancer gene expression in the context of burns and sepsis

We applied the Wilcoxon rank sum test (also known as the Mann-Whitney U test), a nonparametric method, to assess gene expression differences between cancerous and normal tissue across multiple cancer types (44, 45). This test was chosen for its suitability with non-normally distributed data, unlike parametric alternatives that require normal distribution assumptions. Statistical significance was established at α = 0.05, indicating meaningful divergence between the median values of two independent sample groups. We analyzed de-identified gene expression data, represented as transcripts per million (TPM), from The Cancer Genome Atlas (TCGA) for tumor samples and late-stage normal tissue samples from the Genotype-Tissue Expression (GTEx) project. These datasets were accessed via the UCSC Xena database. To mitigate inter-dataset variability, the gene expression values were normalized using Z-scores, allowing for consistent dimensional comparisons. Our primary analysis centered on the glioblastoma multiforme (GBM) dataset, through which we investigated gene expression disparities between cancerous and non-cancerous tissues. This detailed comparison allowed us to observe exercise-induced changes in gene expression, providing insights into potential therapeutic implications for managing burns and sepsis within the cancer context.

### Promoter methylation analysis of genes linked to exercise impacts on burns and sepsis

This study provides an overview of methylation levels across diverse genomic regions, including the TSS1500 region (200–1500 bp upstream of the transcription start site), the proximal promoter region (the first 200 bp upstream from the TSS), the first exon, and the 5’ untranslated region (UTR) (46, 47). For each sample, median methylation values within these regions were determined to represent the sample’s overall methylation status. To investigate the correlation between methylation levels and gene expression, we applied Spearman’s rank correlation, a non-parametric test ideal for assessing relationships between variables that may not exhibit linear patterns. In this analysis, methylation levels served as the independent variables, while gene expression levels were treated as dependent variables. The strength of these associations was quantified using the Spearman rank correlation coefficient. Additionally, we employed the Wilcoxon rank sum test to compare methylation levels between tumor and normal tissue samples. This non-parametric test is appropriate for comparing two independent sample groups without assuming a specific data distribution, thereby enabling a reliable distinction between tumor and normal tissue methylation patterns across different sample sources. Through this approach, we identified significant methylation differences, enhancing our understanding of tumor biology and the potential role of exercise in modulating responses to burns and sepsis.

### ATAC-seq analysis of exercise-related gene modulation in burns and sepsis

In this study, we employed ChIPseeker, an R package tailored for analyzing and visualizing both ChIP-seq and ATAC-seq data (48, 49). Utilizing ChIPseeker’s annotatePeak function, we conducted an in-depth analysis of transcription start sites (TSS) within promoter regions of genes. We set the parameter tssRegion = c (-3000, 3000) to cover the 3000 base pairs upstream and downstream of the TSS, enabling a comprehensive examination of genomic elements, including transcription factor binding sites and histone modifications. To visualize coverage, we utilized ChIPseeker’s covplot function, generating plots that illustrate the distribution of peaks across the genome in human ATAC-seq data. These coverage plots reveal not only the spatial distribution of peaks along chromosomes but also offer detailed insights, including gene names, tumor types, chromosomal positions, and genetic distances, conveniently displayed on the left side of the graph. This visual approach provides researchers with a holistic and precise representation of the ATAC-seq data, enhancing our understanding of exercise-induced gene regulation in the context of burns and sepsis.

### Prognostic assessment of exercise-related genes in burns and sepsis across pan-cancer

To evaluate the prognostic significance of various genes, we applied a univariate Cox regression model using the survival package in R (50, 51). For each gene, we derived hazard ratios along with their 95% confidence intervals by fitting the data into a Cox proportional hazards model through the coxph() function. The results were visualized in a heatmap to facilitate comparative analysis and enhance interpretability.

### Genomic profiling of exercise-related genes in burns, sepsis, and pan-cancer contexts

To investigate the impact of exercise on genes associated with burns and sepsis across various cancer types, we analyzed copy number alterations and DNA methylation data from The Cancer Genome Atlas (TCGA) (52, 53). Patient samples were organized in a structured matrix format, with rows representing samples and columns representing genes or genomic regions. After quality control steps to exclude low-quality samples and probes, data were standardized to minimize technical variation. Using tools like GISTIC and CNAnorm, we identified and categorized genomic amplification and deletion events, quantifying their frequencies across the genome. DNA methylation levels at gene promoter regions were assessed on the UALCAN platform, comparing differences between normal and cancerous tissues to elucidate the influence of exercise on wound healing and infection responses. For methylation pattern analysis in specific cancer-associated genes, we utilized the ‘gene visualization’ module available in MethSurv. To further understand genomic impacts, Mutation Annotation Files (MAF) from TCGAbiolinks and tumor mutation burden (TMB) calculations via maftools allowed us to explore links between these genomic features and immunotherapy responsiveness. Statistical analyses, including correlation and survival analyses, were conducted on copy number alteration, DNA methylation, and TMB data to assess their associations with exercise-modulated gene expression in wound healing and infection. These analyses aimed to evaluate the potential impact of these genomic variations on tumor progression and patient outcomes.

### Gene set enrichment analysis across pan-cancer types

This study utilized data from The Cancer Genome Atlas (TCGA) repository, incorporating tumor and normal tissue samples from a specific cancer type (54, 55). The data underwent stringent quality control processes, during which invalid samples and probes were excluded to ensure robustness. Following quality filtering, normalization was applied to mitigate technical variability inherent in the dataset, including adjustments for background noise, often present in raw files. We employed the “limma” package within the R environment, which facilitates background correction, normalization, and statistical analyses for identifying genes with significant differential expression. The criteria for selection involved both log2 fold change (log_2_FC) and p-values. Log_2_FC quantified the relative changes in gene expression, while the p-value assessed statistical significance. For gene set enrichment analysis (GSEA), we used the “clusterProfiler” package in R to annotate and visualize enriched pathways associated with differentially expressed genes in public databases such as KEGG, GO, and Reactome. We relied on the Enrichment Score (ES), which ranges from 0 to 1, as a metric to determine the relevance of pathway alterations in relation to gene expression changes. Finally, to visualize the findings, we utilized R packages, including “ggplot2,” to create various graphical representations. This included simple bar charts, scatter plots, and heatmaps, allowing a clear interpretation of the results. This methodological framework provides a structured approach to interpreting the molecular implications of gene expression variations in cancer.

### Cell culture

Human umbilical vein endothelial cells (HUVECs) were seeded in culture flasks and maintained in Dulbecco’s modified Eagle medium (DMEM, low glucose), supplemented with 10% fetal bovine serum (FBS), 1% endothelial cell growth supplement, and 1% penicillin-streptomycin solution. The cells were incubated at 37°C in a humidified chamber with 5% CO₂ under normoxic conditions. To establish an *in vitro* model, HUVECs were subsequently exposed to 1 μg/mL of lipopolysaccharide (LPS). Eight hours before the experiments, cells were cultured in serum-free DMEM. For differentiation, THP-1 cells were treated with 100 ng/mL phorbol 12-myristate 13-acetate (PMA) for five days to induce macrophage-like characteristics. RAW 264.7 mouse macrophages (ATCC, Rockville, MD, USA) were cultured in DMEM containing 10% heat-inactivated FBS, 100 U/mL penicillin, and 100 μg/mL streptomycin, and incubated at 37°C in a 5% CO₂ atmosphere.

### Cell viability assay

HUVECs, seeded at a density of 5 × 10³ cells per well, were transfected with MLIP knockdown, overexpression, or control plasmids and plated in 96-well plates to assess proliferation. After an initial 16-hour incubation, the cells were either stimulated with LPS or left untreated for 0, 6, and 24-hour intervals. Cell proliferation was measured using the Cell Counting Kit-8 (CCK-8; Beyotime, Beijing, China, Cat#C0038), in accordance with the manufacturer’s protocol. Absorbance was recorded at 450 nm using a BioTek microplate reader (BioTek, U.S.A.).

### Real-time reverse transcription polymerase chain reaction

Total RNA was isolated from the three experimental groups using Trizol reagent (Invitrogen, USA). The isolated RNA was reverse-transcribed into complementary DNA (cDNA) using the PrimeScript II 1st Strand cDNA Synthesis Kit (Takara, Shiga, Japan). Real-time RT-PCR was performed with the SYBR Premix Ex Taq II (Takara, Shiga, Japan) on a StepOnePlus Real-Time PCR system (Applied Biosystems, CA, USA). The relative mRNA expression levels were quantified using the 2^−ΔΔCt method, with GAPDH as the internal control.

### Measurement of oxidative stress and LDH release assay

Cytotoxicity was assessed by quantifying lactate dehydrogenase (LDH) release using a commercial assay kit, following the manufacturer’s instructions. Briefly, 50 μL of supernatant from each well was collected and incubated with reduced nicotinamide adenine dinucleotide (NADH) and pyruvate for 15 minutes at 37°C. The reaction was terminated with the addition of 0.4 mol/L NaOH. LDH activity was measured by recording the absorbance at 440 nm on a SpectraMax M2 spectrophotometer (Molecular Devices, Sunnyvale, CA, USA) and expressed as U/g protein. Additionally, oxidative stress markers, including superoxide dismutase (SOD) and glutathione peroxidase (GSH-Px) activities, along with reactive oxygen species (ROS) and malondialdehyde (MDA) levels, were measured using commercially available kits, following the manufacturer’s protocols.

### Flow cytometry analysis

Apoptosis and cell polarization were evaluated in both treated and untreated cells using flow cytometry. Following treatment, cells were harvested and stained with FITC-Annexin V and propidium iodide (PI) for 10 minutes at room temperature, following the protocol provided by the Annexin V-EGFP Apoptosis Detection Kit (KeyGEN BioTECH). For polarization assessment, cells were additionally stained with antibodies targeting M1 polarization markers when required. Data acquisition and analysis were conducted on a Fluorescence-Activated Cell Sorting (FACS) Calibur flow cytometer (Becton-Dickinson, Sparks, MD, USA) with associated software. For the colony formation assay, cells were plated in 6-well plates and treated with designated drugs for 6 hours according to the experimental groups for reactive oxygen species (ROS) detection. A fluorescent probe, DCFH-DA (10 µM; Solarbio, China), was diluted in a serum-free medium, and cells were incubated with it for 30 minutes at 37°C. FITC signals were subsequently detected via flow cytometry.

### Immunofluorescence analysis

Cells were plated in 6-well culture plates and incubated overnight to ensure adherence. The cells were then fixed with 3.7% paraformaldehyde at room temperature for 15 minutes and subsequently permeabilized in cold methanol at -20°C for 15 minutes. Blocking was performed using a buffer containing 5% normal goat serum and 0.5% Triton X-100 in PBS for 1 hour at room temperature. Primary antibodies were added, and cells were incubated overnight at 4°C. After washing three times with PBS for 10 minutes each, cells were incubated at room temperature with Alexa Fluor 488-conjugated goat anti-rabbit secondary antibody (Cat# A-11034) and Alexa Fluor 594-conjugated goat anti-mouse secondary antibody (Cat# A-11004; Thermo Fisher), both diluted 1:500 in blocking buffer. Prior to imaging, nuclei were stained with DAPI (Cat# D9542; Sigma) for 30 minutes at room temperature. Images were obtained using a Nikon Eclipse E800 fluorescence microscope.

### Statistical analysis

Data are presented as mean ± standard deviation (SD). Statistical analyses were conducted using GraphPad Prism 8 software. Group differences were assessed using either Student’s t-test or analysis of variance (ANOVA), depending on the experimental design. A p-value of less than 0.05 (P < 0.05) was considered statistically significant.

## Results

### Gene expression and pathway enrichment analysis

**Figure 1:**
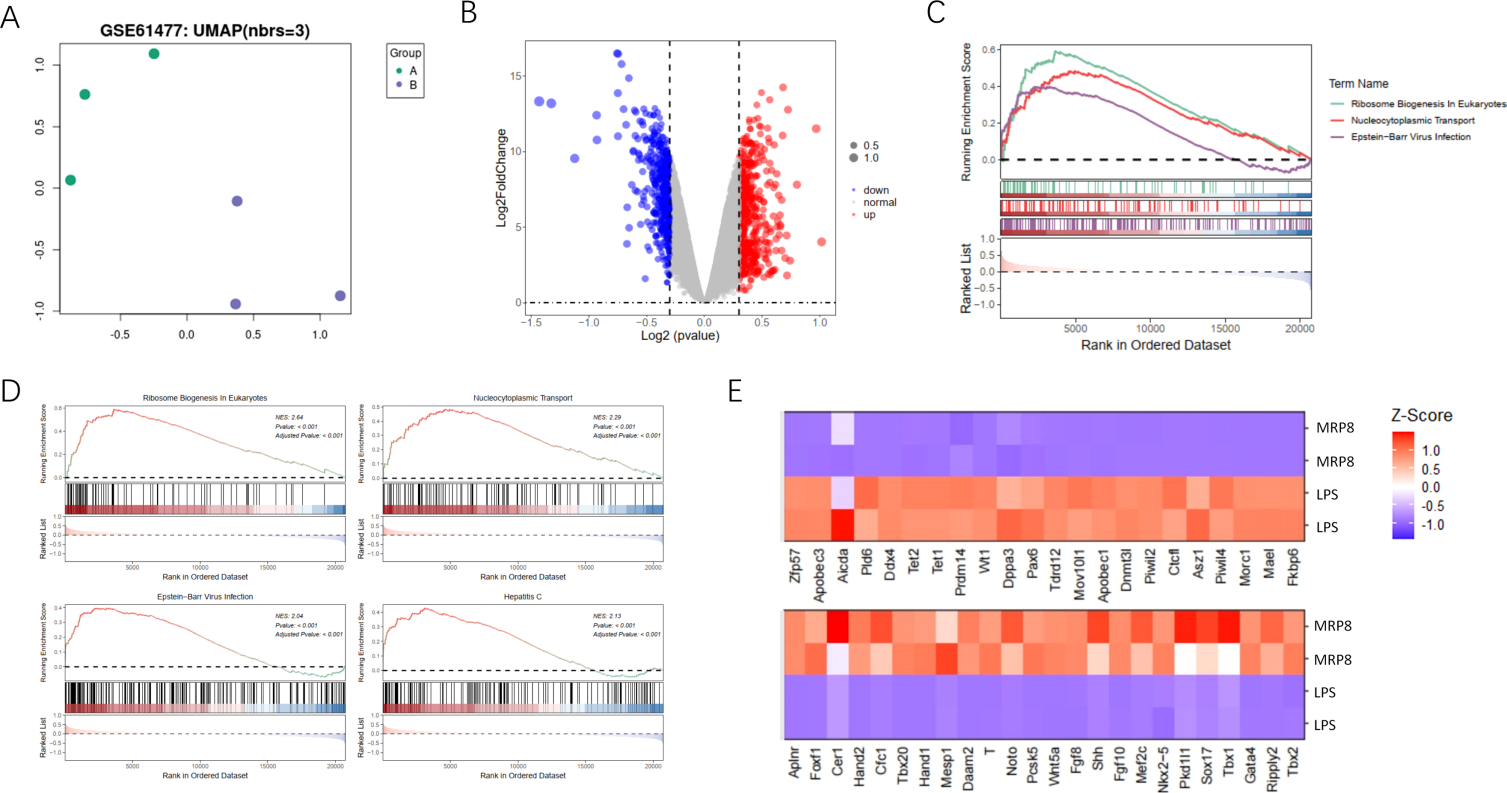
Analysis of gene expression and pathway enrichment between two sample groups. **(A)** The UMAP plot shows the two-dimensional distribution of the two sample groups visualized using Uniform Manifold Approximation and Projection (UMAP). Each point represents an individual sample, color-coded by group, and illustrates clustering patterns based on gene expression profiles. **(B)** The volcano plot presents differential gene expression analysis between the two groups. The x-axis displays the log2 fold change in gene expression between groups, and the y-axis shows the -log10 adjusted p-value to indicate statistical significance. Red points highlight significantly upregulated genes, blue points represent significantly downregulated genes, and grey points indicate genes without significant changes. **(C)** The GSEA plot illustrates gene set enrichment analysis, highlighting pathways with significant enrichment. The enrichment score is plotted across the rank-ordered gene list for three specific pathways: Immune Response in Activation, Metabolic Pathways, and Tumor Suppressor Inhibition, each represented by different colors and line types. **(D)** The GSEA top pathways display the four most significantly enriched pathways individually through GSEA. Each panel includes an enrichment score curve, indicating the positions of genes within the pathway on the ranked gene list, revealing the distribution and relevance of genes contributing to pathway enrichment. **(E)** The heatmap visualizes the expression levels of genes identified in the enriched pathways. Each row represents a specific gene, and each column corresponds to a sample. Gene expression levels are normalized to z-scores, with red indicating high expression and blue indicating low expression, allowing a visual comparison of expression patterns across samples in each group.

In our study, we investigated gene expression differences and pathway enrichment between two sample groups using a variety of analytical approaches. The UMAP plot (**Figure 1A**) revealed distinct clustering between the groups, indicating significant differences in gene expression profiles. This clustering suggests that the gene expression profiles of the groups are sufficiently divergent to merit further investigation into the underlying molecular mechanisms. The volcano plot (**Figure 1B**) highlighted differentially expressed genes. This visualization not only underscores the extent of differential expression but also aids in the identification of key genes that may be crucial in the context of burn-induced sepsis and its potential association with cancer. GSEA identified significantly enriched pathways (**Figure 1C**), including those related to immune response activation, metabolic pathways, and tumor suppressor inhibition. The top four enriched pathways (**Figure 1D**) were further explored, with the enrichment scores and the specific positions of genes within these pathways clearly displayed. A heatmap (**Figure 1E**) illustrates the Z-scores of gene expression for genes within these pathways, providing a clear visualization of expression patterns across all samples. This comprehensive analysis highlights significant differences in gene expression and pathway enrichment between the two groups, emphasizing key pathways involved in disease mechanisms and pinpointing potential therapeutic targets. These findings lay the groundwork for identifying therapeutic targets and biomarker candidates, which could play a crucial role in the development of more effective treatment strategies for burn-induced sepsis and its potential link to cancer.

### Differential gene expression and pathway enrichment analysis

**Figure 2:**
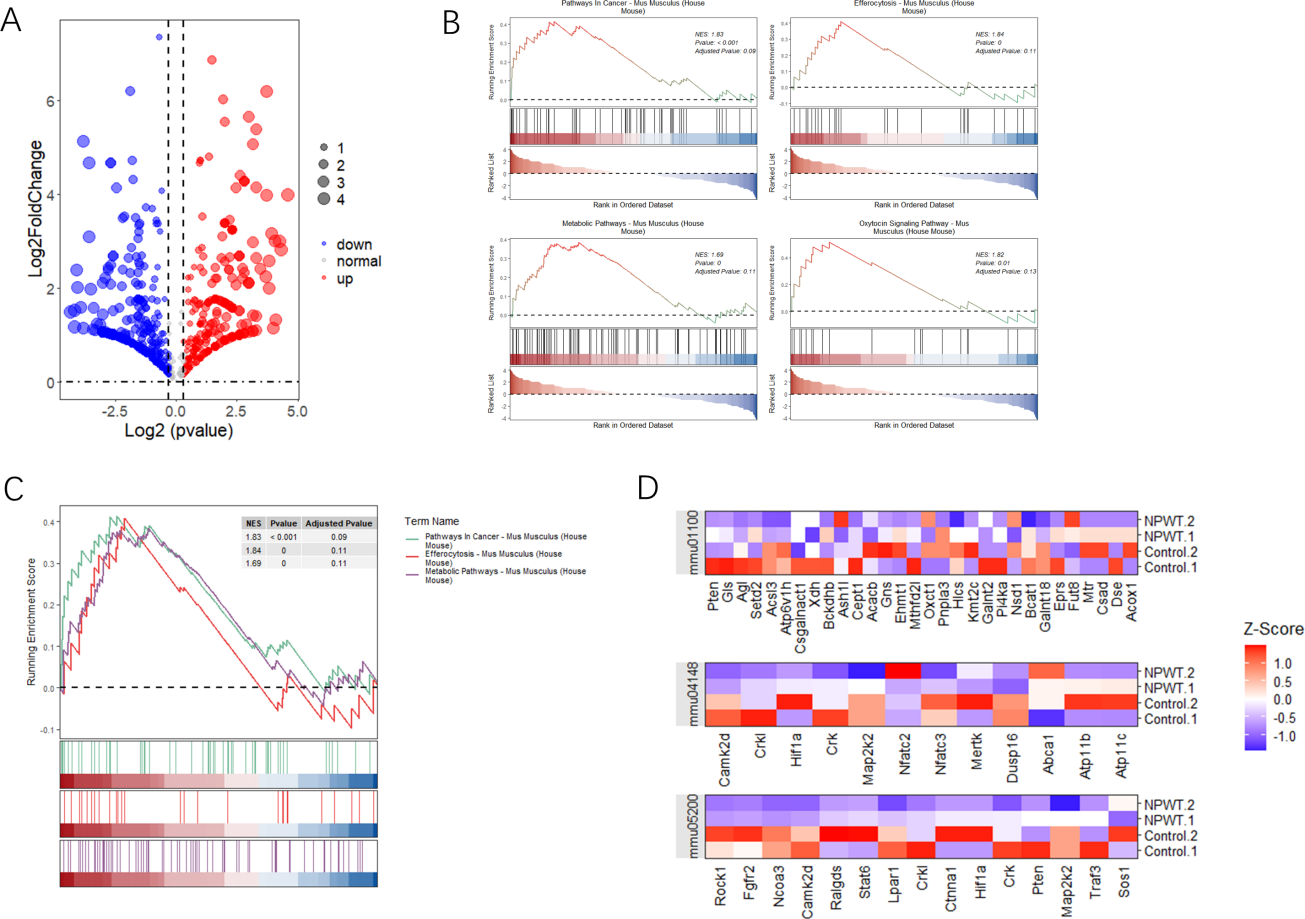
Analysis of differential gene expression and pathway enrichment. **(A)** The volcano plot illustrates the differential expression of genes between two groups. The x-axis represents log2 fold change, while the y-axis shows -log10(p-value). Red highlights genes with significant upregulation, blue indicates downregulated genes, and dashed lines mark the thresholds for statistical significance. **(B)** Gene Set Enrichment Analysis (GSEA) plots illustrate the enrichment of three pathways with significant results. The y-axis displays the enrichment score (ES), and the x-axis shows the rank of genes within the ordered dataset. Key pathways enriched in this analysis are emphasized based on their statistical significance. **(C)** Additional GSEA plots present the top four pathways with notable enrichment scores, further emphasizing the relevance of pathways that show substantial statistical enrichment. **(D)** A heatmap shows the expression levels of genes across significant pathways, normalized by Z-score across all samples. Each row represents a gene, and each column represents a sample, with color gradation indicating expression levels—red for high expression and blue for low. This comparison includes gene expression data from both tumor and normal tissue samples, allowing a comparative analysis of gene expression under different conditions.

Our study aimed to identify differentially expressed genes (DEGs) and significantly enriched pathways between tumor and normal tissue samples. To achieve this, we utilized a combination of statistical and computational tools, with the results presented in various figures to provide clarity and a comprehensive understanding of the findings. **Figure 2A** presents a volcano plot that illustrates the distribution of DEGs between tumor and normal groups. The dashed lines represent thresholds for statistical significance, enabling the clear identification of the most relevant genes affected by the conditions under investigation. GSEA was conducted to identify significantly enriched pathways, with **Figure 2B** depicting the statistical significance of these pathways. This figure reveals the biological processes most influenced by the differential gene expression patterns observed between tumor and normal tissues. The pathways selected based on their statistical significance offer valuable insights into the biological processes that are most affected by the differentially expressed genes. Further GSEA results highlighted the top four enriched pathways, as shown in **Figure 2C**. Additionally, the gene distribution within these pathways is shown, offering a clearer understanding of how specific genes contribute to pathway enrichment and their potential role in tumor development. **Figure 2D** displays a heatmap that visualizes gene expression levels across all samples, specifically focusing on genes related to the identified pathways. Each row in the heatmap corresponds to a unique gene, while each column represents an individual sample. Collectively, these analyses provide a detailed view of the molecular alterations occurring in tumor and normal tissues. The identification of significantly enriched pathways and differentially expressed genes contributes to a deeper understanding of the biological functions and pathways involved in tumorigenesis.

### Expression landscape of exercise-influenced genes in pan-cancer analysis

**Figure 3:**
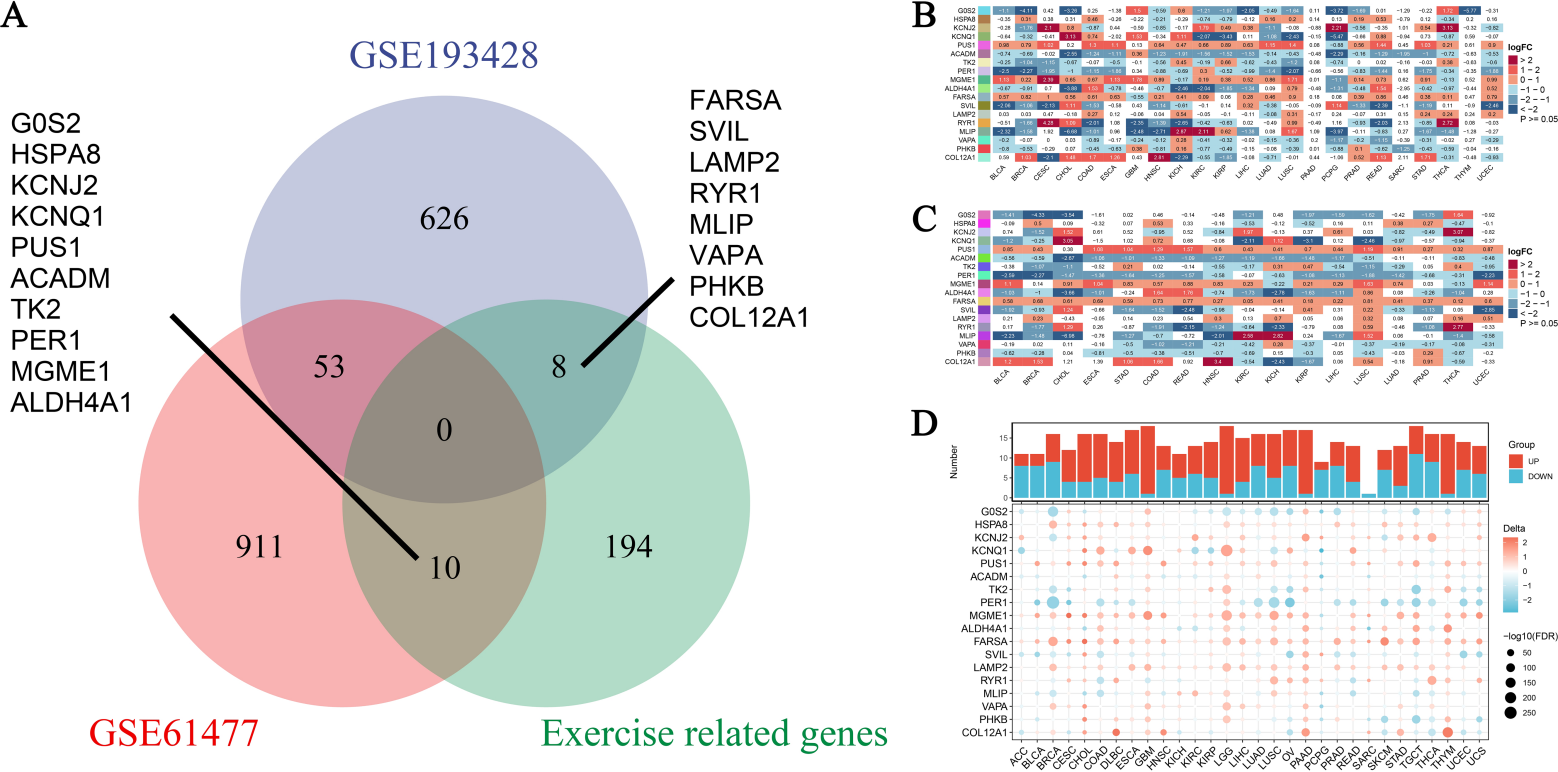
Expression profiles of exercise-modulated genes related to burns and sepsis in pan-cancer analysis. **(A)** The Venn diagram illustrates the overlap of differentially expressed genes (DEGs) identified from two datasets, GSE193428 and GSE61477, including a subset of genes associated with exercise. This visualization distinguishes DEGs unique to each dataset from those shared between them, underscoring genes commonly modulated by exercise, burns, and sepsis. Specific genes unique to each condition are labeled outside the Venn circles. **(B)** Differential expression analysis was conducted on exercise-modulated genes linked to burns and sepsis using non-paired samples across multiple cancer types. The resulting heatmap depicts the expression levels of these genes, with color intensity reflecting the extent of expression change. The data, drawn from various cancer types, showcases gene expression variations influenced by exercise, burns, and sepsis. **(C)** A subsequent differential expression analysis was performed on the same gene set, using paired samples from individual patients. This heatmap presents refined insights into gene expression changes by comparing tumor tissue directly with adjacent normal tissue from the same patient. Analyzing paired samples helps control for individual variability, revealing more consistent gene expression patterns related to exercise influences. **(D)** Additional differential expression analysis incorporates data from the TCGA-GTEx projects, providing a comprehensive comparison of gene expression across cancerous and normal tissues. This analysis examines exercise’s influence on genes associated with burns and sepsis. At the top, a bar chart details the number of samples analyzed, while the heatmap below indicates shifts in gene expression, with color coding denoting increased or decreased levels.

In this study, we analyzed the expression landscape of exercise-influenced genes associated with burns and sepsis. The datasets used included GSE193428, GSE61477, and a dataset of exercise-related genes, collectively providing a comprehensive profile of gene expression. By integrating data from these diverse sources, we aimed to investigate the shared and unique gene expression patterns influenced by exercise in the context of burns, sepsis, and cancer. As shown in **Figure 3A**, a Venn diagram illustrates the overlap and unique characteristics of DEGs across the datasets. Specifically, 626 genes were unique to GSE193428, 911 were specific to GSE61477, and 194 were exclusive to the exercise-related genes. Additionally, 53 genes overlapped between GSE193428 and GSE61477, while 10 genes were common between GSE61477 and the exercise-related gene dataset. Key genes identified among these, such as FARSA, SVIL, LAMP2, and COL12A1, were found to be influenced by exercise in both burns and sepsis, suggesting their potential roles in exercise-mediated recovery processes. These genes are involved in critical biological functions such as protein synthesis, cell signaling, and tissue repair, all of which are vital for enhancing the body’s response to burns, sepsis, and potentially cancer development. **Figures 3B, C** present the differential expression analysis of these genes across various cancer types. **Figure 3B** illustrates the expression levels using non-paired samples, capturing the variability in gene expression due to exercise, burns, and sepsis. In contrast, **Figure 3C** employs paired samples to compare tumor and adjacent normal tissues, providing a more refined analysis that controls for individual variability and highlights consistent exercise-influenced expression patterns. Expanding on these analyses, **Figure 3D** integrates data from both the TCGA and GTEx projects, enabling a comprehensive comparison of gene expression profiles in malignant and healthy tissues. This figure offers further insights into how physical activity may modulate expression changes associated with burns and sepsis. At the top of **Figure 3D**, the sample sizes for each cancer type are displayed. Below, a heatmap uses color coding to represent upregulated and downregulated genes, highlighting the expression patterns across cancer types. These findings underscore the significant influence of exercise on transcriptional patterns associated with burns and sepsis in the context of cancer. The consistent expression changes observed in exercise-influenced genes across various datasets suggest that exercise may be a critical modulator of recovery from both sepsis and burns, with potential implications for cancer prevention and survival.

### Analysis of promoter methylation of burn and sepsis-related genes influenced by exercise

**Figure 4:**
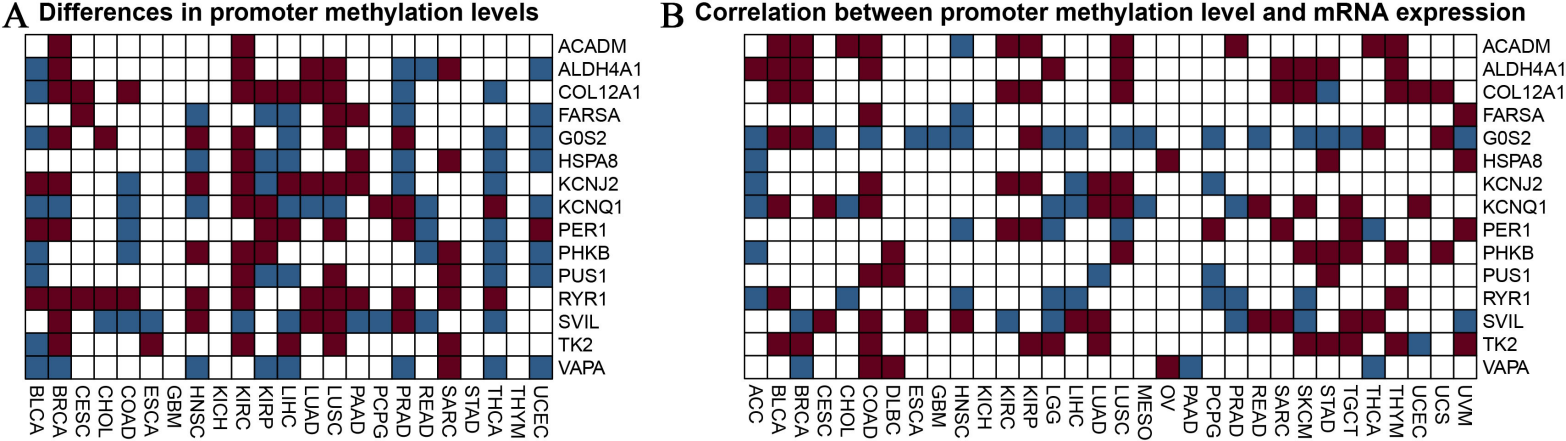
Analysis of promoter methylation in exercise-modulated genes related to nurns and sepsis. **(A)** The heatmap visualizes differences in promoter methylation for specific genes associated with burns and sepsis, including ACADM, ALDH4A1, COL12A1, FARS2, G6PC, HSP90A, KCNJ2, KCNQ1, PER1, PHKB, PUS1, RYR1, SVIL, TK2, and VAPA. The colors in the heatmap represent methylation levels, with red indicating hypermethylation, blue indicating hypomethylation, and white showing no significant change. This analysis compares samples from patients receiving exercise treatment with those who did not. **(B)** The heatmap examines the correlation between promoter methylation and mRNA expression levels for the same set of genes. It visually represents the relationship between changes in methylation and mRNA expression, using the same color scheme as in panel **(A)**. Red indicates a negative correlation, blue indicates a positive correlation, and white shows no significant correlation. This analysis seeks to understand how variations in promoter methylation impact gene expression.

**Figure 4** provides an in-depth analysis of promoter methylation levels and their impact on mRNA expression in burn- and sepsis-related genes affected by physical activity. **Figure 4A** presents a heatmap that displays variations in promoter methylation levels for genes such as ACADM, ALDH4A1, COL12A1, FARS2, G6PC, HSP90A, KCNJ2, KCNQ1, PER1, PHKB, PUS1, RYR1, SVIL, TK2, and VAPA. In this heatmap, red represents hypermethylation, blue indicates hypomethylation, and white denotes no significant change. The analysis compares methylation patterns across patient samples, distinguishing those who underwent exercise intervention from those who did not, revealing notable differences that suggest the influence of exercise on the epigenetic regulation of these genes. **Figure 4B** illustrates the relationship between promoter methylation levels and corresponding mRNA expression levels, employing the same color scheme: red for a negative correlation, blue for a positive correlation, and white for no significant correlation. This heatmap demonstrates how changes in promoter methylation can impact gene expression, with hypermethylation generally associated with reduced mRNA expression and hypomethylation linked to increased expression levels. The analysis was conducted with rigorous statistical evaluations, examining parameters such as distribution, mean, median, standard deviation, and variance to ensure objectivity and precision in data interpretation. These findings highlight the potential regulatory role of exercise in gene expression related to burn and sepsis recovery, emphasizing the significance of considering epigenetic modifications in therapeutic strategies and suggesting the potential benefits of exercise interventions in clinical settings. Future research should aim to further elucidate the mechanisms behind these epigenetic modifications and their implications for recovery in burn and sepsis. Investigating how specific exercise interventions might influence gene methylation pathways and understanding their functional consequences on gene expression will provide deeper insights into the potential therapeutic benefits of exercise. This analysis provides valuable insights into the influence of exercise on promoter methylation and gene expression, making a substantial contribution to the growing field of exercise genomics and its role in medical treatments.

### Promoter methylation analysis of burn and sepsis-related genes affected by exercise

Promoter methylation analysis revealed varying methylation patterns across burn and sepsis-related genes influenced by exercise. ACADM showed mainly unmethylated or low methylation, with variable CpG site methylation (**Supplementary Figure 1A**). ALDH4A1 had predominantly unmethylated sites (**Supplementary Figure 1B**), while COL12A1 exhibited low to medium methylation hotspots (**Supplementary Figure 1C**). FARSA had a balanced methylation pattern with some sites highly methylated (**Supplementary Figure 1D**). G0S2 showed low methylation levels with specific CpG site variation (**Supplementary Figure 1E**). HSPA8 and LAMP2 were primarily lowly methylated (**Supplementary Figures 1F, I**). KCNJ2 and KCNQ1 exhibited medium to high methylation (**Supplementary Figures 1G, H**). MGME1 and SVIL had balanced methylation with significant site-specific methylation (**Supplementary Figures 1J, P**). MLIP showed varied methylation, notably high levels (**Supplementary Figure 1K**), while PER1 and PHKB had low to medium methylation (**Supplementary Figures 1L, M**). PUS1 had medium to high methylation (**Supplementary Figure 1N**), and RYR1 was largely unmethylated (**Supplementary Figure 1O**). TK2 mainly exhibited low methylation (**Supplementary Figure 1Q**), and VAPA was mostly unmethylated or lowly methylated (**Supplementary Figure 1R**). These results highlight exercise’s potential role in regulating the epigenetic modification of these genes, providing insight into the underlying molecular mechanisms and potential therapeutic applications.

### Correlation of burn and sepsis-related gene expression with tumor prognosis

**Figure 5:**
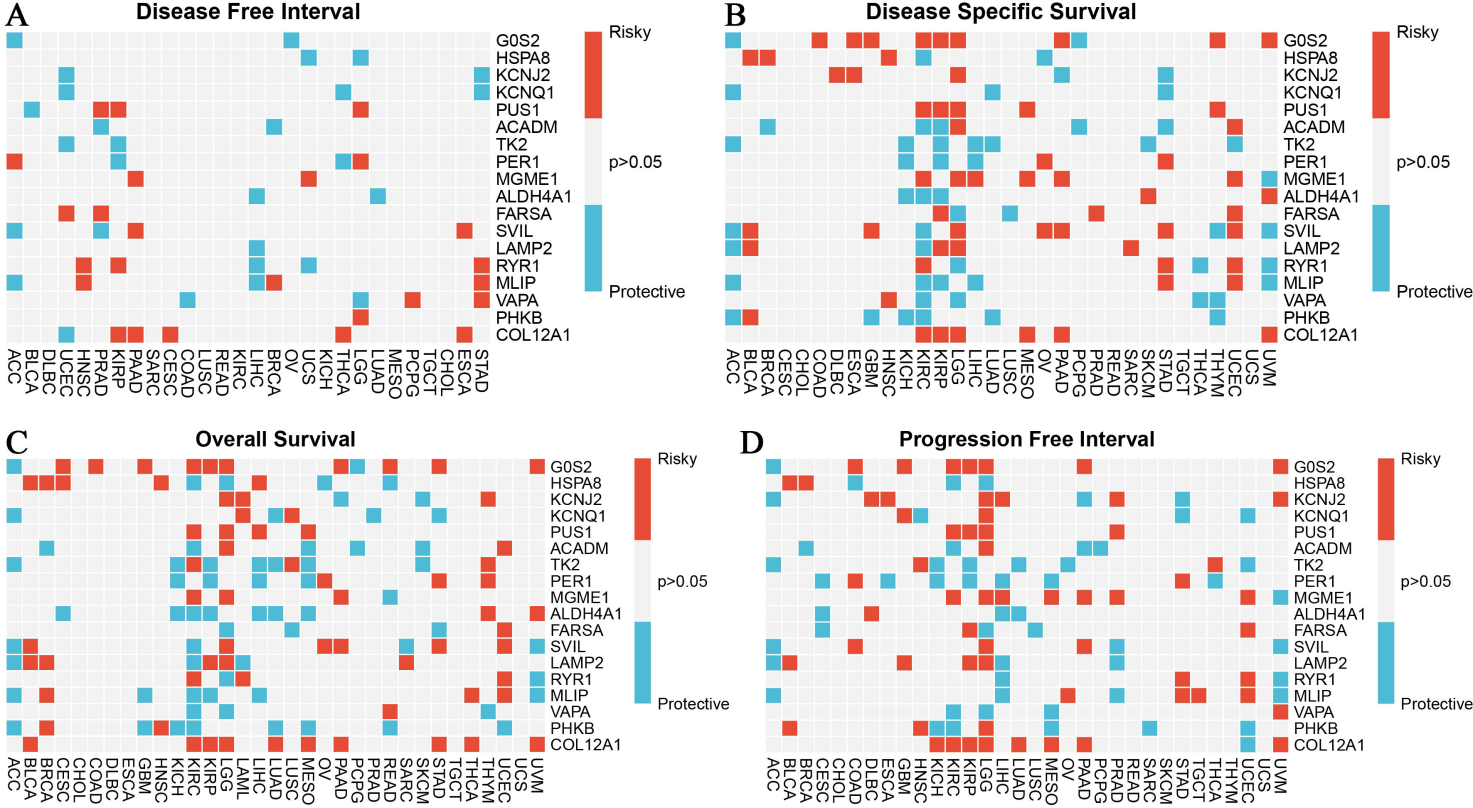
Association of burn and sepsis-related gene expression with tumor prognosis indicators (DFI, DSS, OS, PFI). **(A)** The heatmap shows the correlation between the expression of burn and sepsis-associated genes and the disease-free interval in cancer patients. Red squares represent genes correlated with an increased risk (risky), while blue squares indicate genes with a protective effect. Only significant correlations (p<0.05) are shown. **(B)** The heatmap illustrates the relationship between gene expression and disease-specific survival in cancer patients, following the same color coding as in panel **(A)**. Red squares denote risky genes, and blue squares indicate protective genes, with only significant associations (p<0.05) included. **(C)** This panel provides a heatmap that demonstrates the correlation between gene expression and overall survival in cancer patients. Genes associated with a higher risk are shown in red, while those with protective associations are in blue. Only significant correlations (p<0.05) are highlighted. **(D)** The heatmap examines the correlation between gene expression and progression-free interval in cancer patients. As in the previous panels, red indicates risky genes, and blue represents protective genes. Only statistically significant correlations (p<0.05) are displayed.

Our study examined the correlation between the expression of burn- and sepsis-related genes and tumor prognosis, specifically focusing on Disease-Free Interval (DFI), Disease-Specific Survival (DSS), Overall Survival (OS), and Progression-Free Interval (PFI) (**Figure 5**). In the Disease-Free Interval (DFI) panel (**Figure 5A**), a heatmap displays the relationship between gene expression and DFI in tumor patients. Here, red squares denote genes associated with increased risk, while blue squares indicate protective genes, with significant correlations marked (p < 0.05). Similarly, the Disease-Specific Survival (DSS) panel (**Figure 5B**) presents a heatmap that illustrates the correlation between gene expression and DSS, maintaining the same color scheme and significance threshold. The Overall Survival (OS) panel (**Figure 5C**) shows correlations between gene expression and OS, with red indicating risky genes and blue representing protective genes. Only statistically significant correlations (p < 0.05) are displayed to emphasize meaningful relationships. Lastly, the Progression-Free Interval (PFI) panel (**Figure 5D**) provides a heatmap showing the correlation between gene expression and PFI, using consistent color coding and significance criteria. These findings highlight the significant influence of specific gene expressions on various tumor prognosis metrics, offering valuable insights that could guide the development of potential therapeutic targets. The study emphasizes the importance of considering the molecular landscape shaped by burn and sepsis-related genes, which may provide novel opportunities for personalized treatments in cancer patients, particularly those recovering from burns or sepsis.

### Comprehensive analysis of exercise-influenced burn and sepsis-related genes across pan-cancer

This study explores the impact of exercise on genes associated with burns and sepsis across various cancer types, revealing several key findings. **Supplementary Figure 2A** shows mutation frequencies of these genes in 20 cancer types, with color intensity representing mutation rates. A waterfall plot (**Supplementary Figure 2B**) highlights mutation variability across categories. **Figures 2C, D** illustrate the correlation between gene expression and Tumor Mutation Burden (TMB), with bubble sizes indicating correlation significance and colors reflecting the strength and direction. **Figures 2E, F** categorize samples by copy number amplifications, identifying the most common cancer types. **Figure 2G** presents cumulative copy number alterations, while **Figure 2H** compares amplifications and deletions across cancer types. Finally, **Figure 2I** displays a correlation matrix of gene expression in different cancers, with bubble sizes representing p-values. These findings highlight the heterogeneous genomic effects of exercise on burn- and sepsis-related genes in cancers, offering insights into potential therapeutic targets and informing personalized cancer treatment strategies.

### Exercise-influenced gene expression in cancer and microbiomes

**Figure 6:**
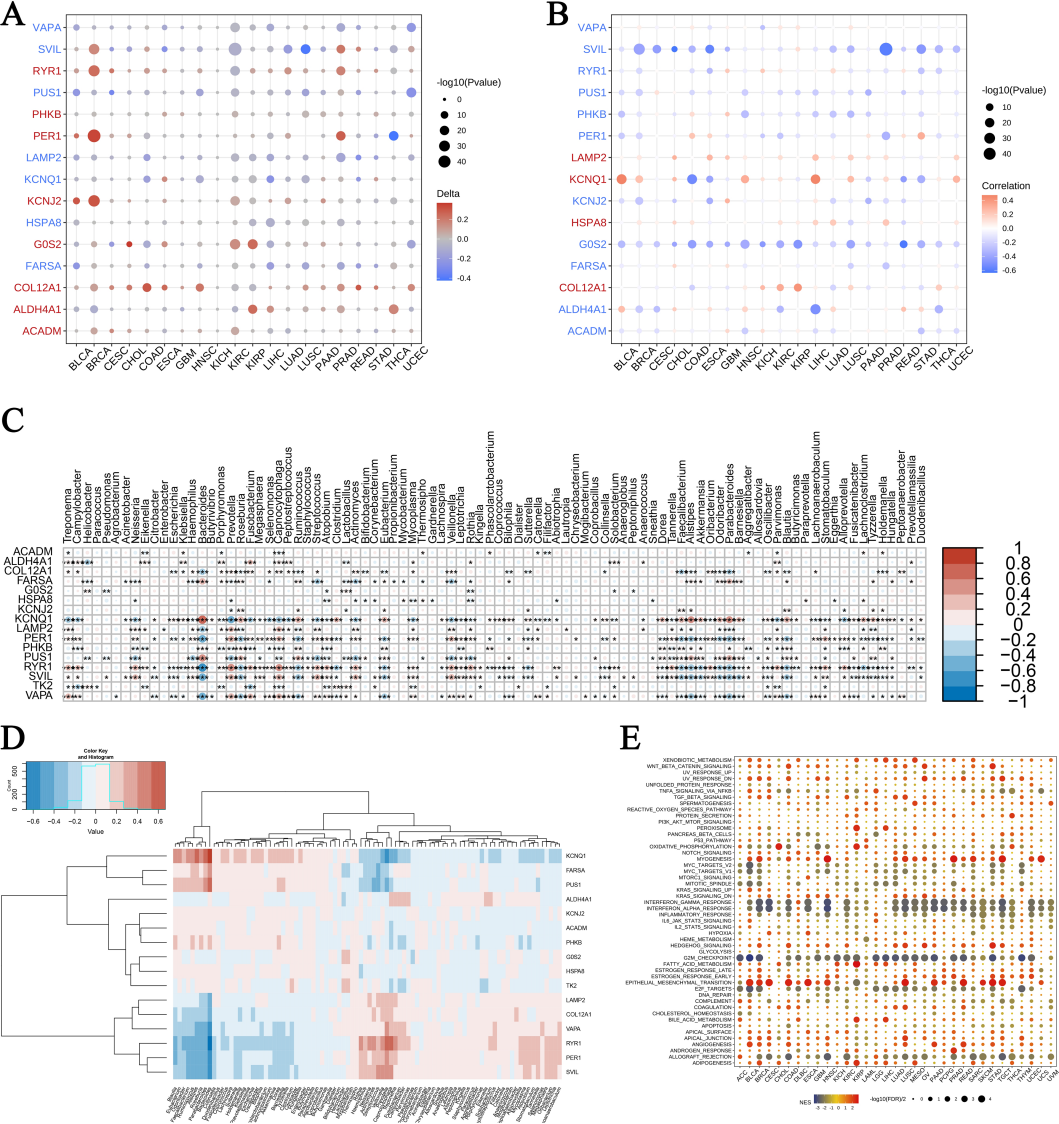
Pan-cancer enrichment analysis and gene expression in microbiomes. **(A)** Association of exercise-influenced burn and sepsis gene expression with CNV in multiple cancer types: This panel presents the correlation between the expression of exercise-modulated genes associated with burns and sepsis and copy number variations (CNV) across various cancer types. Circle size corresponds to the significance level (-log10 p-value), while the color gradient indicates delta values, with red representing upregulation and blue indicating downregulation. **(B)** Association of gene expression with promoter methylation across cancer types: This panel displays the correlation between gene expression and promoter methylation levels for exercise-influenced genes related to burns and sepsis across multiple cancers. Circle size reflects the significance level (-log10 p-value), while the color gradient denotes correlation values, with blue indicating negative correlation and red showing positive correlation. **(C, D)** Expression of exercise-influenced genes in microbiomes: The heatmap in panel **(C)** illustrates expression levels of these genes across various microbiomes, with color intensity representing normalized expression levels. Panel **(D)** depicts hierarchical clustering of gene expression data, with a color scale from blue (low expression) to red (high expression), showing distinct clustering patterns. **(E)** GSEA pathway enrichment analysis in different cancers: This bubble plot illustrates the pathways enriched in different cancers, with bubble size representing the number of genes involved and color indicating the enrichment score—red for a higher enrichment score and blue for a lower score. The symbols *, **, and *** represent statistical significance levels corresponding to p<0.05, p<0.01, and p<0.001, respectively.

Our analysis of gene expression following exercise across multiple cancer types revealed notable enrichments in genomic features and microbiome signatures. In **Figure 6A**, we observed distinctive gene expression patterns associated with burns and sepsis that correlate with CNVs. In this figure, circle sizes denote significance levels, while color gradients indicate delta values, highlighting differential expression. Similarly, **Figure 6B** shows the relationship between gene expression and promoter methylation, with blue representing negative correlations and red indicating positive correlations. **Figures 6C, D** explore the expression of these genes within different microbiomes. A heatmap in **Figure 6C** presents normalized expression levels, while hierarchical clustering in **Figure 6D** reveals co-regulated gene clusters influenced by microbial presence. **Figure 6E** provides a GSEA across various cancers, identifying enriched pathways. Bubble sizes correspond to the number of genes involved, and colors reflect enrichment scores, with red indicating higher scores. These findings suggest that exercise modulates gene expression in ways that affect cancer biology and microbial interactions, offering potential insights for therapeutic development. The interaction between exercise, gene expression, and microbiome modulation highlights new avenues for improving cancer treatment strategies through holistic approaches that consider genetic, epigenetic, and microbial factors.

### Pan-cancer GSVA enrichment analysis

This study performed an extensive pan-cancer analysis of burn- and sepsis-related gene sets influenced by exercise across various cancer types using four scoring methods: combined z-scores, GSVA z-scores, PLAGE z-scores, and ssGSEA z-scores. Results in **Supplementary Figure 3** reveal significant differences in gene set enrichment between normal and tumor tissues. **Supplementary Figure 3A** presents the combined z-scores, showing notable statistical significance in cancers such as KIRC and BLCA, which suggests differential gene expression. **Supplementary Figure 3B** displays the GSVA z-scores, with marked variations in THCA and KICH, pointing to potential implications for tumor biology. In **Supplementary Figure 3C**, the PLAGE z-scores highlight significant differences in KIRP and BLCA, underscoring the influence of exercise on gene set activity. Finally, **Supplementary Figure 3D** shows the ssGSEA z-scores, revealing substantial changes across multiple cancers, suggesting therapeutic potential for exercise-modulated genes in cancer treatment. This analysis provides key insights into the role of exercise-related genes in cancer biology, emphasizing their potential therapeutic applications. It opens avenues for future research, particularly in identifying cancer types that could benefit from exercise-based interventions and exploring the molecular mechanisms underlying exercise-induced gene expression changes in cancer.

### MLIP modulates inflammation, oxidative stress, and macrophage polarization in LPS-induced RAW264.7 cells

**Figure 7:**
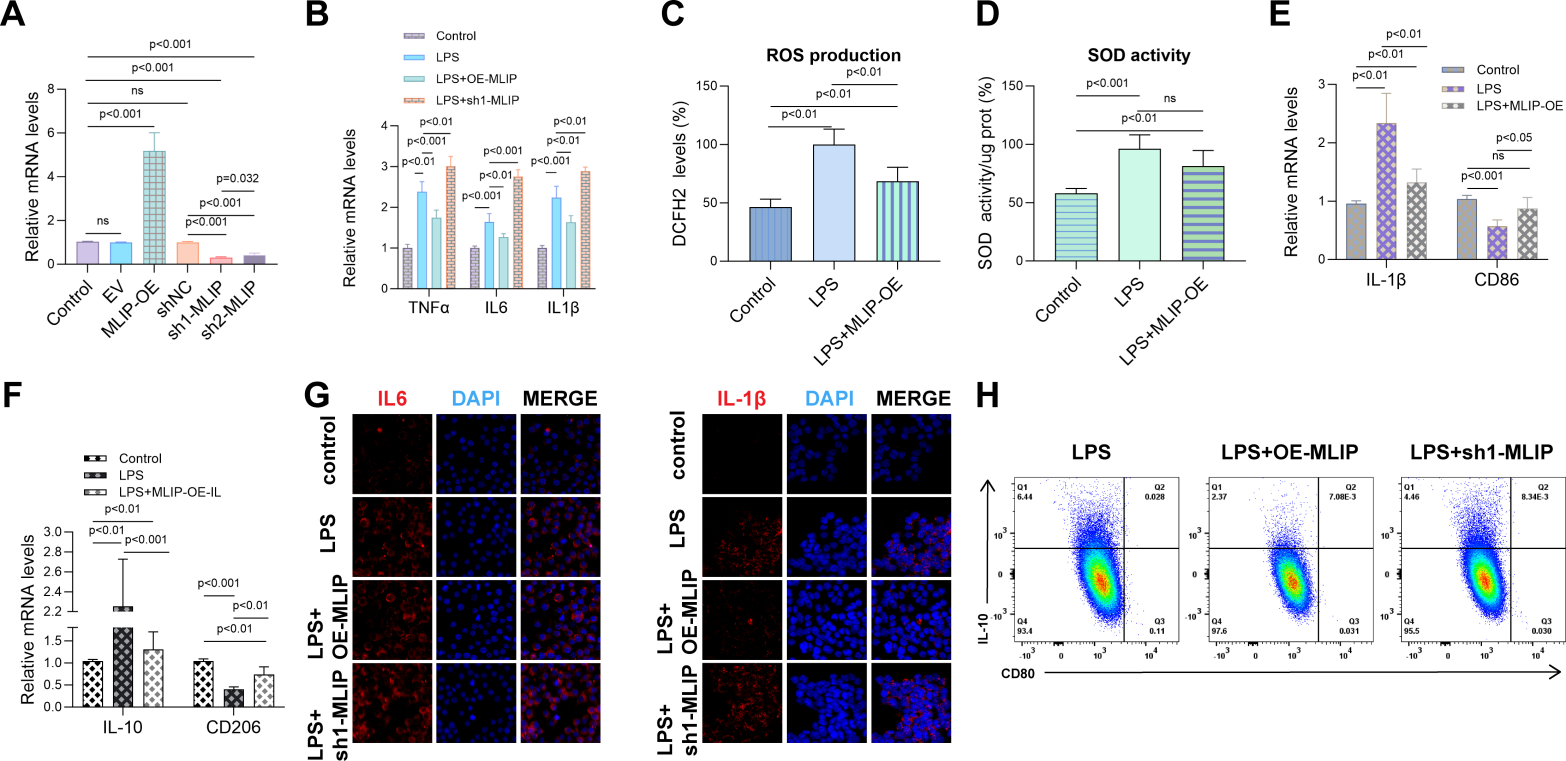
MLIP’s role in modulating inflammation, oxidative stress, and polarization in LPS-induced RAW264.7 cells. **(A)** Quantitative RT-PCR analysis of target gene expression in RAW264.7 cells across various treatment groups, including Control, MLIP overexpression (MLIP-OE), MLIP knockdown (sh-MLIP), and LPS-stimulated cells. Expression levels are normalized to the Control group and reported as mean ± SD, with p < 0.05 indicating statistical significance. **(B)** Quantitative RT-PCR analysis showing relative mRNA levels of TNF-α, IL-6, and IL-1β in Control, LPS, LPS + MLIP-OE, and LPS + sh-MLIP groups, demonstrating MLIP’s role in modulating inflammation in the context of LPS treatment. **(C)** DCFH-DA assay results illustrate the effects of different treatments on intracellular reactive oxygen species (ROS) levels. The Control group serves as the baseline, while LPS treatment induces ROS production, and LPS + MLIP-OE treatment reduces ROS, suggesting an antioxidative role for MLIP. **(D)** Measurement of SOD activity across Control, LPS, and LPS + MLIP-OE groups. LPS significantly reduces SOD activity, while MLIP overexpression restores SOD function in treated cells. Data are expressed as mean ± SD, with p < 0.05 indicating statistical significance. **(E)** qRT-PCR analysis of M1 macrophage markers IL-1β and CD86 in Control, LPS, and LPS + MLIP-OE groups, highlighting MLIP’s influence on macrophage polarization. **(F)** Comparison of IL-10 and CD206 expression levels in Control, LPS, and LPS + MLIP-OE groups using qRT-PCR. Data are shown as mean ± SD, with p < 0.05 denoting statistical significance. **(G)** Immunofluorescence staining of inflammatory cytokines: Representative immunofluorescence images illustrating IL-6 and IL-1β expression in RAW264.7 cells across Control, LPS, LPS + MLIP-OE, and LPS + sh-MLIP groups. Cells are stained with DAPI (blue) to label nuclei, with IL-6 or IL-1β (red) indicating cytokine localization and expression. **(H)** Flow cytometry analysis of macrophage polarization: Flow cytometric plots showing the impact of LPS, LPS + MLIP-OE, and LPS + sh-MLIP treatments on macrophage polarization, measured by surface marker expression. Data illustrate shifts in polarization states influenced by MLIP modulation. The results represent three independent experiments. ns, not significant.

This study investigated the role of MLIP in regulating inflammation, oxidative stress, and macrophage polarization in LPS-induced RAW264.7 cells. **Figure 7A** shows that MLIP overexpression (MLIP-OE) significantly enhanced the expression of target genes, while MLIP knockdown (sh-MLIP) notably reduced expression, highlighting MLIP’s regulatory function. **Figure 7B** reveals that LPS treatment upregulated TNF-α, IL-6, and IL-1β mRNA levels, indicating an intensified inflammatory response. MLIP-OE attenuated these cytokine levels, suggesting its potential to suppress inflammation, while LPS + sh-MLIP further elevated inflammatory markers, indicating that MLIP inhibition exacerbates inflammation. **Figure 7C** demonstrates increased ROS production in the LPS group, reflecting heightened oxidative stress. MLIP-OE reduced ROS levels compared to LPS treatment alone, indicating that MLIP mitigates oxidative stress, while sh-MLIP further increased ROS levels. **Figure 7D** illustrates that superoxide dismutase (SOD) activity, a key antioxidant enzyme, was reduced by LPS but partially restored with MLIP-OE, underscoring the antioxidative role of MLIP. **Figures 7E, F** explore MLIP’s effects on macrophage polarization. MLIP-OE reduced the expression of the M1 marker CD86 and IL-1β, while increasing the expression of the M2 marker CD206, suggesting MLIP’s role in promoting a balanced macrophage polarization. IL-10 levels remained highest in the LPS group, reflecting the influence of LPS on anti-inflammatory responses. **Figure 7G**, through immunofluorescence staining, shows reduced IL-6 and IL-1β expression in the MLIP-OE group compared to the LPS and sh-MLIP groups, further supporting MLIP’s anti-inflammatory effects. Finally, **Figure 7H** presents flow cytometry data showing shifts in macrophage polarization profiles: MLIP-OE promoted a less inflammatory state, while sh-MLIP favored pro-inflammatory polarization. Overall, these results highlight the significant role of MLIP in modulating inflammation, oxidative stress, and macrophage polarization in LPS-induced RAW264.7 cells. These findings suggest that MLIP could serve as a potential therapeutic target for inflammatory diseases, particularly those involving macrophage polarization, such as autoimmune disorders, infections, and chronic inflammatory conditions.

### The role of MLIP in regulating inflammation, oxidative stress, and cell proliferation in HUVECs, with implications for burn-induced sepsis and cancer progression

**Figure 8:**
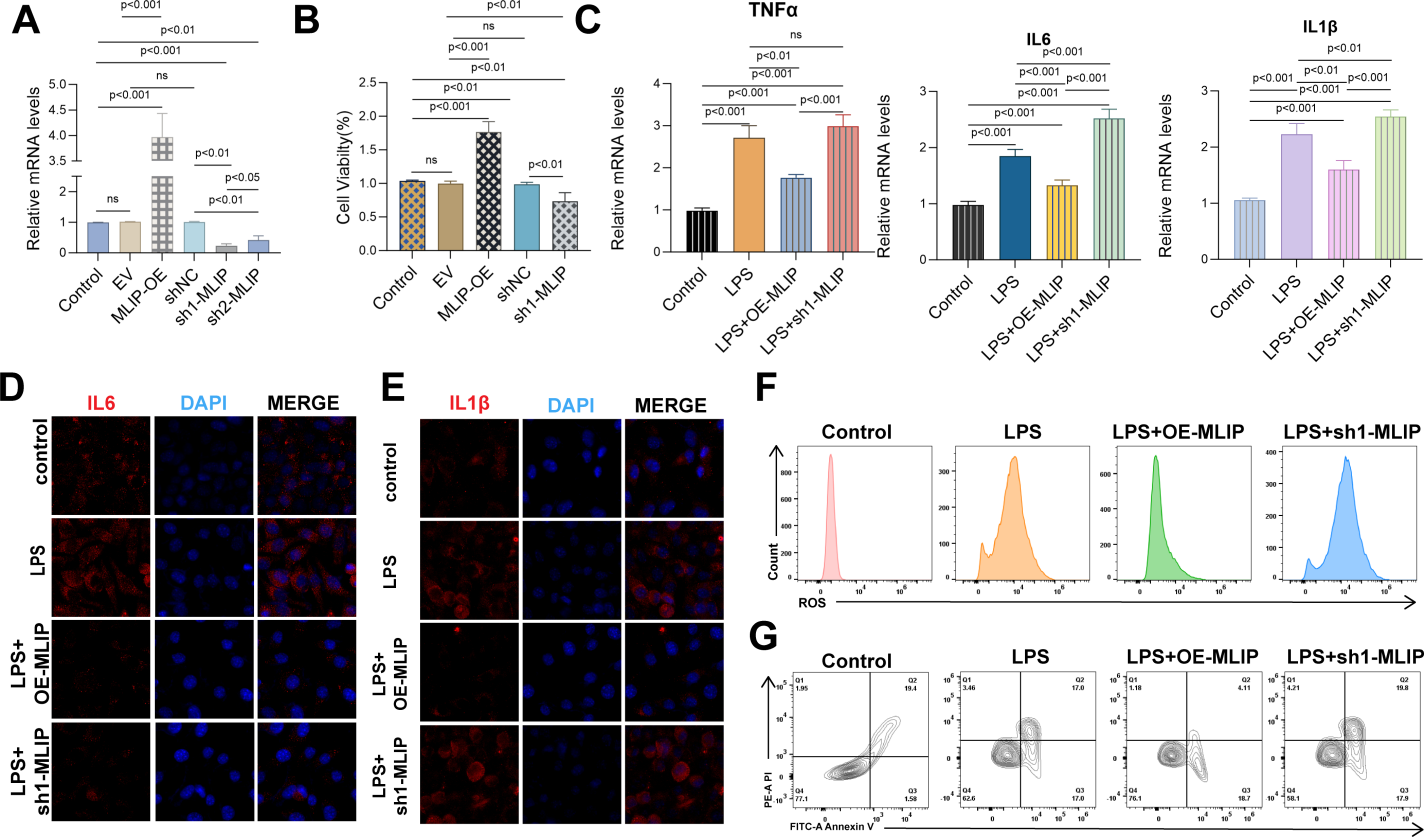
Effects of MLIP on gene expression, cell viability, inflammation, ROS levels, and apoptosis in HUVECs across treatment conditions. **(A)** The relative mRNA expression levels of target genes were analyzed via qRT-PCR across treatment groups, with the Control group as a baseline. The EV (empty vector) group serves as a negative control, MLIP-OE represents MLIP overexpression, and sh-MLIP indicates MLIP knockdown. Data represent mean ± SD from three independent experiments, with *p < 0.05 denoting statistical significance. **(B)** HUVEC proliferation was measured under various conditions. Control represents untreated cells, EV serves as the empty vector control, MLIP-OE represents MLIP overexpression, and sh-MLIP indicates MLIP silencing. Results are expressed as mean ± SD, with *p < 0.05 indicating significant differences. **(C)** mRNA levels of inflammatory cytokines were measured by qRT-PCR in Control, LPS-stimulated, LPS + MLIP-OE, and LPS + sh-MLIP groups. LPS stimulation models inflammation, and subsequent treatments assess MLIP’s role in modulating cytokine expression. Data are presented as mean ± SD, with *p < 0.05 indicating significance. **(D)** Immunofluorescence staining of IL-6 (red) with DAPI (blue) was performed on HUVECs to assess IL-6 expression under different treatment conditions, including LPS stimulation and MLIP modulation. **(E)** Cells were stained for IL-1β (red) and DAPI (blue), revealing IL-1β expression changes due to LPS treatment, MLIP overexpression, and MLIP knockdown. **(F)** Flow cytometry quantified ROS levels in HUVECs under Control, LPS, LPS + MLIP-OE, and LPS + sh-MLIP conditions, highlighting MLIP’s role in regulating oxidative stress during inflammation. **(G)** The percentage of apoptotic cells was measured across treatment groups: Control, LPS-stimulated, LPS + MLIP-OE, and LPS + sh-MLIP. Data underscore MLIP’s influence on LPS-induced apoptosis, illustrating differences in apoptosis rates between groups. ns, not significant.

**Figure 9:**
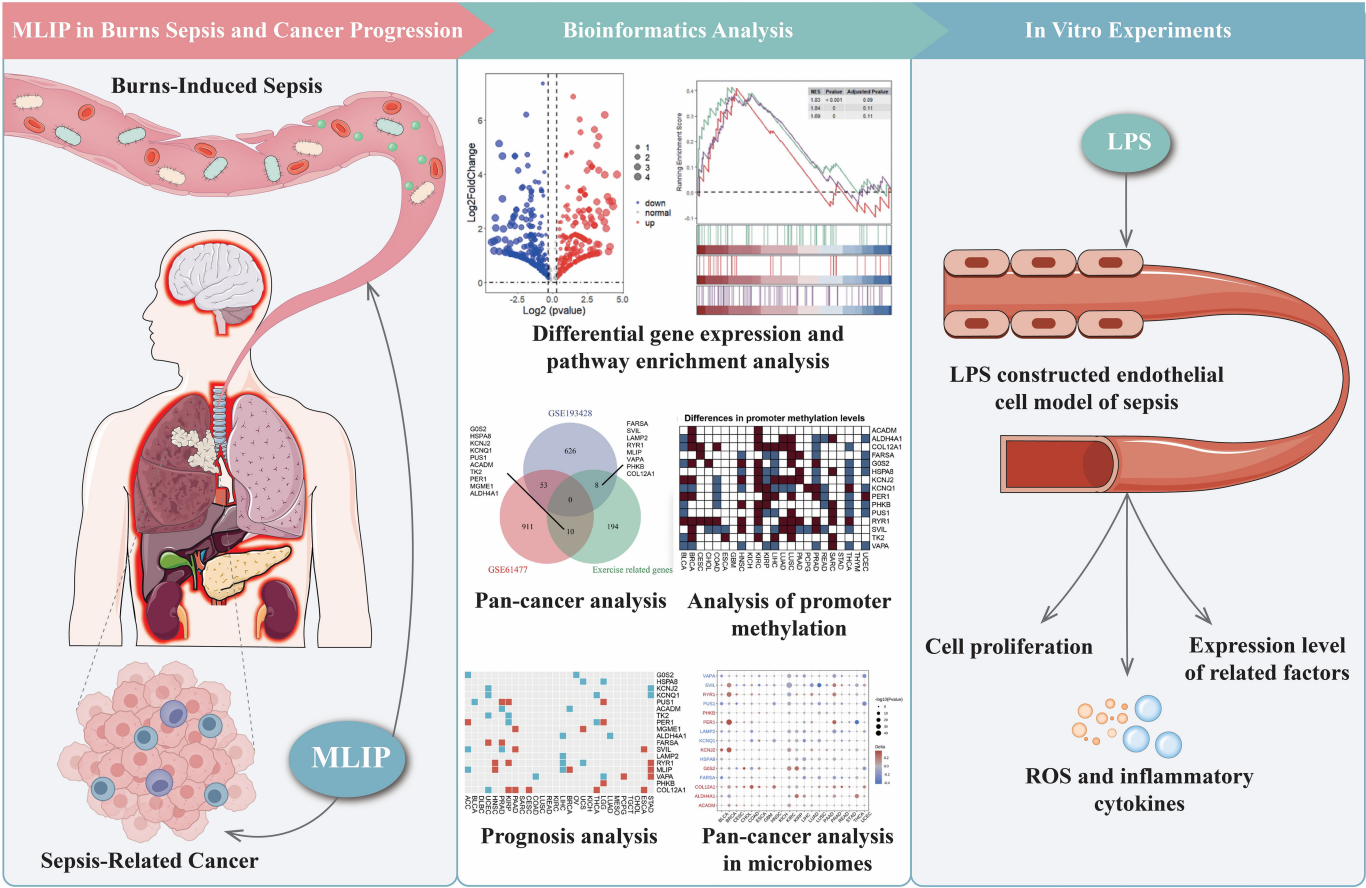
Comprehensive analysis of MLIP’s role in burns-induced sepsis and cancer progression through bioinformatics and *in vitro* studies. *In vivo* findings highlight MLIP’s role in modulating immune responses, suggesting its potential in preventing the transition from sepsis to cancer. The middle section focuses on bioinformatics, analyzing differential gene expression, pathway enrichment, and Gene Set Enrichment Analysis (GSEA) using RNA-seq data. The right section describes *in vitro* experiments using an endothelial cell model of sepsis, where cells are treated with lipopolysaccharide (LPS). These experiments assess cell proliferation, inflammatory markers, ROS, and cytokine levels, shedding light on immune responses during septic conditions. This comprehensive figure explores MLIP’s involvement in burns-induced sepsis and cancer progression, presenting potential therapeutic targets.

This study explores MLIP’s role in modulating gene expression, inflammation, oxidative stress, and cell proliferation in human umbilical vein endothelial cells (HUVECs). Additionally, it investigates MLIP’s potential involvement in burn-induced sepsis and subsequent cancer progression. As illustrated in **Figure 8A**, MLIP overexpression markedly increased the relative mRNA levels of target genes, whereas MLIP knockdown reduced these levels, underscoring its significant function in gene regulation. **Figure 8B** demonstrates that MLIP overexpression enhances cell viability, as indicated by the CCK-8 assay, while knockdown decreases proliferation, suggesting MLIP’s supportive role in cell growth. Further, **Figure 8C** compares the expression levels of pro-inflammatory cytokines TNFα, IL-6, and IL-1β across different treatments. The LPS + MLIP-OE group displayed lower cytokine levels than the LPS-only group, underscoring MLIP’s potential anti-inflammatory effect. Conversely, increased cytokine levels in the LPS + sh-MLIP group suggest that MLIP inhibition may amplify inflammatory responses. Immunofluorescence analysis of IL-6 and IL-1β (**Figures 8D, E**) corroborates these findings, showing reduced inflammatory marker expression with MLIP overexpression and elevated levels with knockdown. Moreover, flow cytometry analysis of ROS production (**Figure 8F**) reveals that MLIP overexpression alleviates LPS-induced oxidative stress, while knockdown elevates ROS levels, highlighting MLIP’s regulatory influence on oxidative stress. Apoptosis analysis (**Figure 8G**) indicates that MLIP overexpression mitigates LPS-induced apoptosis, whereas its inhibition increases apoptosis rates, suggesting a protective role against cell death. **Figure 9** provides a comprehensive view of MLIP’s involvement in burn-induced sepsis and its implications for cancer progression related to sepsis. The left panel traces the progression from burns to systemic inflammation and sepsis, showing MLIP’s role in immune modulation. Central bioinformatics analysis demonstrates differential gene expression, pathway enrichment, and pan-cancer analysis, elucidating MLIP’s influence on specific pathways and epigenetic regulation during sepsis. Prognostic analysis suggests MLIP’s potential relevance across various cancer types, while microbiome interactions hint at its immunomodulatory properties. The right panel integrates findings from *in vitro* studies on an LPS-induced endothelial model of sepsis, linking MLIP’s effects on cell proliferation, ROS levels, and inflammatory cytokine expression. Collectively, these findings highlight MLIP as a pivotal modulator of cellular responses under inflammatory conditions, offering a foundation for its therapeutic potential in managing sepsis and reducing cancer risk associated with inflammation. These results support the notion that MLIP may act as a central mediator of cellular responses in inflammation, oxidative stress, and immune modulation, with important implications for inflammatory diseases and cancer treatment.

## Discussion

Burn-induced sepsis presents not only a clinical emergency but also a significant public health concern, as supported by epidemiological data. This alarming statistic highlights the critical nature of burn-induced sepsis and underscores the urgent need for enhanced preventive strategies, diagnostic methods, and therapeutic interventions (56, 57). Given macrophages’ pivotal role in immune response, their involvement is central to sepsis pathology. Understanding macrophage-mediated inflammatory responses offers a promising avenue for identifying potential therapeutic targets in treating burn-induced sepsis.

Recent advancements in the medical research field have provided great hope in addressing the complexities of burn-induced sepsis. This study establishes a solid theoretical foundation for improving intervention strategies and discovering new therapeutic targets (58). Given these findings, an appropriate exercise regimen for burn patients could potentially enhance immune function and recovery, thereby reducing susceptibility to sepsis. Significant progress has also been made in nanotechnology. Through bioinformatics, scientists have gained a deeper understanding of the relationship between the microstructure and properties of nanomaterials. This has enabled precise control over pore size, porosity, and composition, leading to broad applications in catalysis, adsorption, and biomedicine (59, 60). For instance, the development of near-infrared light-activated upconversion nanoparticles/curcumin mixed nanomedicines has shown promising potential in inducing glioma stem cell differentiation and effective eradication (61). While this research primarily focuses on gliomas, the concept of using nanomaterials for targeted therapy can be applied to burn-induced sepsis. Researchers have successfully identified potential biomarkers and developed risk prediction models, offering new perspectives for personalized treatment strategies (62–64). Large datasets have also been utilized for bioinformatics analysis, facilitating the precise identification of biomarkers, the exploration of signaling pathways, and the systematic study of immune characteristics (65–67). In the context of burn-induced sepsis, this could lead to early detection through the identification of sepsis-specific biomarkers. Moreover, assessing patients’ immune profiles allows for more accurate prognosis, enabling timely adjustments to treatment strategies.

Our research focuses on gene expression profiles and epigenetic modifications in the context of burn - induced sepsis and cancer. These processes are essential for understanding how sepsis and tumorigenesis are interlinked, as they may facilitate tumor cell proliferation and survival while also potentially enhancing immune evasion (31, 68). For instance, cytokines such as TNF-α and IL-6, produced during sepsis, play a key role in organizing inflammatory responses and may indirectly support tumor formation (31, 69). Additionally, oxidative stress associated with sepsis can result in DNA double-strand breaks and genomic instability, creating a genetic foundation conducive to tumor initiation and progression (70). These findings highlight the need for further exploration into the impact of sepsis on tumor microenvironments, particularly in terms of immune suppression and the initiation of genomic instability, both of which could present novel therapeutic opportunities. Examining how macrophage dysfunction during sepsis contributes to site-specific organ damage may provide novel insights into controlling inflammation and preventing cancer progression, depending on the affected sites. We are investigating how exercise influences gene expression profiles and epigenetic modifications across medical conditions such as burns, sepsis, and cancer (71, 72). The Wilcoxon rank - sum test was applied to analyze gene expression differences between tumor and normal tissues. Sourcing data from relevant databases and performing necessary normalizations, we also analyzed methylation levels in specific genomic regions.

These findings underscore the particular significance and timeliness of our study. By investigating the mechanisms of burn-induced sepsis, we aim to provide deeper insights into this clinical issue from both molecular and biological perspectives (73). Our research contributes not only to the development of new preventative and therapeutic strategies to reduce sepsis risk and improve outcomes for burn patients but also to the understanding of the intricate link between inflammation and tumor development. By expanding scientific knowledge on the connections between burn-induced sepsis and tumorigenesis, we can provide valuable data to support informed policy development, potentially leading to measurable reductions in the incidence and healthcare costs of these diseases on a broader scale (13, 31).

This study employed a multitude of approaches to explore the therapeutic potential of molecular targets, with bioinformatics playing a pivotal role in data interpretation. From a microscopic perspective of molecules and cells, the combination of transcriptomics and proteomics has delved deep into disease mechanisms, revealing the crucial regulatory mechanisms of transcription factor networks and protein modifications in diseases. This has laid a theoretical foundation for developing novel treatment strategies (74). Through the combined analysis of metabolomics and proteomics, the metabolic pathways and their significant roles in cell functions have been explored multiple times (75). In cell - level research, an in - depth exploration of cell polarization and its role in immune regulation and treatment strategies has provided important evidence for understanding cell functions and immune regulation mechanisms (76). The advancements in proteomics technology have enabled a more thorough study of protein - protein interaction networks, their modifications, and regulatory mechanisms. This helps uncover the complex signal transduction processes within cells, laying the groundwork for understanding the essence of life activities and developing new targeted treatment strategies (77, 78). Through bioinformatics analysis, key genes associated with specific diseases were identified, and their roles in immune infiltration were explored, indicating the direction for disease diagnosis and the search for treatment targets (79, 80). Notably, the inclusion of bioinformatics tools in the analysis has provided insights into the molecular underpinnings of burn-induced sepsis and their connections to cancer-related pathways. Bioinformatics tools were applied to annotate gene functions and conduct pathway enrichment analyses, revealing potential signaling pathways and regulatory networks involved in burn - induced sepsis and its connection to cancer - related pathways. *In vivo* studies using animal models further substantiated these findings. For instance, RNA-seq allows researchers to quantify gene expression across various tumor types in comparison to normal tissues, identifying gene expression profiles associated with cancer development (81, 82). High - throughput sequencing technologies, like RNA - seq, combined with bioinformatics analysis, were used to investigate gene expression profiles in tumor and normal tissues, identifying profiles associated with cancer development.

Bisulfite sequencing (BS-seq) and chromatin accessibility assays offer insights into epigenetic regulatory mechanisms by inferring the DNA methylation status of gene promoters. This is crucial for understanding epigenetic modifications that lead to the activation or silencing of transcription start sites (TSS) (83, 84). Additionally, ATAC-seq technology identifies open chromatin regions that may interact with transcription factors and other regulatory proteins, playing a vital role in controlling gene expression (85, 86). We anticipate that future work will further explore how these epigenetic modifications could potentially serve as therapeutic targets in burn-induced sepsis and cancer. This approach may ultimately assist in identifying novel biomarkers for early cancer detection, particularly in individuals who have experienced burn-induced sepsis. GSEA can be applied to immune-related gene sets, providing an integrated perspective on immune regulatory mechanisms within the tumor microenvironment (87). Additionally, microbial differential expression studies enable the examination of gene expression profiles within microbial communities in tumor contexts, shedding light on the relationships between microbes, tumor development, and treatment responses (88).

This study systematically investigated the role of MLIP in RAW264.7 cells and HUVECs through *in vitro* experiments, with a particular focus on MLIP’s regulation of inflammation. Initially, PCR was first utilized to assess MLIP’s effects on gene expression in RAW264.7 cells and HUVECs, while the CCK - 8 assay evaluated cell proliferation (89, 90). Subsequently, an inflammatory response model was established by treating cells with LPS, allowing us to observe the regulatory effects of MLIP on inflammatory markers, including TNFα (tumor necrosis factor-alpha) and IL-6 (interleukin-6), as well as its impact on cellular processes like hydration and electrical conductivity during inflammation (91, 92). Our experiments highlighted MLIP’s potential in regulating immune responses, offering a promising strategy for modulating inflammation in burn-induced sepsis. Additionally, flow cytometry was used to examine MLIP’s regulatory influence on M1 and M2 macrophage polarization (93). This comprehensive experimental approach aims to elucidate the underlying mechanisms by which MLIP may influence inflammatory diseases, offering theoretical support for its potential therapeutic target (94). The insights gained into macrophage polarization and ROS modulation suggest possible pathways for targeted interventions in the management of sepsis-related organ damage.

Through a comprehensive analysis of data from the GEO database, we identified a cohort of genes associated with burn and sepsis that are influenced by exercise and exhibit distinct expression patterns in various tumor samples compared to normal tissues (95, 96). Returning to the macroscopic perspective of clinical applications and disease research, when evaluating treatment efficacy and prognostic indicators, multiple factors such as metabolic tumor burden and immune cell characteristics were comprehensively considered, providing a multi - dimensional perspective for judging disease progression and treatment responses (97, 98). In studying the associations between diseases and other factors, methods such as Mendelian randomization studies and case - control studies play important roles. For example, exploring the association between cholecystectomy and the risk of a certain disease, as well as comparing the molecular characteristic differences of different disease onset types (99). Animal models were used to verify *in-vivo* effects, and histological and immunohistochemical analyses were carried out to observe tissue pathological changes, providing important evidence for subsequent research (80). Mendelian randomization analysis was applied to explore the causal relationship between metabolites and diseases. This method effectively eliminates the influence of confounding factors and improves the accuracy of causal inference (100). While focusing on disease treatment, the humanistic care for patients should not be overlooked. Peer support and patient participation have a significant effect on improving the treatment experience and quality of life of cancer patients (101). Spiritual beliefs play an important psychological support role in patients at the end - stage of diseases (102). Further investigation focused on the methylation status of the promoter regions of these genes. We observed substantial changes in methylation levels in tumor cells compared to normal cells. Notably, increased methylation of specific genes was associated with their silencing, potentially serving as a mechanism for tumor cells to evade immune surveillance and facilitate tumor progression (103, 104). Using ATAC-seq technology, we assessed the chromatin accessibility of these genes. The results indicated that chromatin regions associated with tumor progression were more accessible in tumor cells, allowing transcription factors and other regulatory proteins to bind more easily, thereby modulating gene expression (105, 106). These findings underscore the potential of macrophage-targeted therapies to alter the tumor microenvironment and improve patient outcomes in sepsis-associated cancers. In the broader context of medical research, significant advancements have been made in various related fields. In materials science, breakthroughs in nanomaterial preparation have opened up broad prospects in fields such as catalysis, adsorption, and biomedicine (107, 108). In analyzing core genes across various cancers, we examined copy number variations, methylation status, and tumor mutation burden, uncovering complex genetic and epigenetic alterations (109, 110). Specifically, mutations in key genes associated with tumor aggressiveness and resistance to chemotherapy were identified, suggesting new strategies for developing targeted therapies against these genes (111, 112). GSEA further indicated an enrichment of these core genes in immune-related pathways, highlighting their possible roles in modulating the tumor immune microenvironment. Additionally, differential expression analysis of these genes across various microbiomes suggested their involvement in host-microbe interactions within the tumor microenvironment (113).

This study highlights the significant biological functions of MLIP in RAW264.7 cells and HUVECs. In RAW264.7 cells, MLIP overexpression markedly increased gene expression as assessed by PCR, underscoring its crucial role in gene regulation. Following LPS treatment, the expression of inflammatory markers TNFα, IL-6, and IL-1β was significantly elevated; however, these levels were relatively lower in the MLIP overexpression group, suggesting that MLIP may play a role in suppressing inflammatory responses. Conversely, the elevated inflammatory factors observed in the MLIP knockdown group imply that MLIP deficiency may exacerbate inflammation. Simultaneously, changes in ROS levels indicated that LPS enhances oxidative stress, while MLIP overexpression appears to moderate ROS levels, highlighting its potential antioxidant function. In terms of M1 macrophage polarization, MLIP expression was closely associated with variations in CD86 and IL-1β levels, further supporting its regulatory role in macrophage polarization. In HUVECs, MLIP overexpression also promoted gene expression and cell proliferation, as demonstrated by CCK-8 assay results. Following LPS treatment, inflammatory factor expression increased significantly, but this rise was tempered in the MLIP overexpression group, suggesting that MLIP helps maintain cell function by modulating inflammatory responses under prolonged stress conditions. Additionally, MLIP was shown to increase cell proliferation rates, as indicated by flow cytometry analysis. Its role in regulating cell polarization and proliferation provides a theoretical foundation for potential therapeutic applications in various inflammatory diseases. MLIP’s regulatory influence extends to the polarization and proliferation of multiple cell types, including RAW264.7 cells and HUVECs. Using the Chickseeker package, we evaluated the spatial proximity of transcription factor binding sites and histone modifications relative to transcription start sites.

MLIP expression was elevated in the MLIP-OE group using an overexpression vector, whereas, in the sh-MLIP group, MLIP expression was suppressed through shRNA targeting (114, 115). Preliminary findings suggest that MLIP may play a critical role in macrophage polarization and metabolic reprogramming, though further studies are needed to elucidate the complex intracellular pathways influenced by MLIP and their impact on macrophage function in both normal and pathological states. A significant amount of data on RNA levels and cellular responses in various conditions was gathered using standardized quantification methods, providing insights into the dual role of MLIP in regulating cell proliferation. Flow cytometry was used to analyze the rate of cell apoptosis, ROS levels, and differences in cell surface markers between the groups. We paid special attention to apoptosis induced by LPS to evaluate whether MLIP confers a protective effect in this pathway. These methodologies clarify MLIP’s role in modulating macrophage activity and its implications in inflammatory responses (116, 117).

This study, through bioinformatics analysis and *in vitro* cell experiments, has demonstrated the pivotal role of MLIP in burn - induced sepsis. Our findings highlight MLIP’s potential as a therapeutic target, as alterations in its expression are closely associated with inflammatory responses and cellular damage. Future research will focus on delineating the specific mechanisms by which MLIP influences sepsis progression, including its roles in inflammatory pathways and effects on cell survival and function. Additionally, we plan to assess the feasibility of therapeutically targeting MLIP, offering new insights and directions for clinical intervention (118, 119). The application of network pharmacology and experimental verification methods in traditional Chinese medicine research has opened up new avenues and strategies for drug development (120). The development of bioinformation technology has promoted breakthroughs in natural product research. The use of molecular network technology for rapid screening and target molecule discovery has effectively accelerated the drug development process (121). Molecular dynamics simulation and structural biology methods are used to analyze the binding characteristics and functions of target proteins, providing important evidence for new drug design (122). Further studies will also explore the collective roles of these genes in tumorigenesis, particularly focusing on their interactions with immune cells within the tumor microenvironment (123, 124). Investigating how these genes respond to exercise and other lifestyle factors may contribute to advancements in personalized medicine (125, 126). We also plan to assess the feasibility of therapeutically targeting MLIP. However, it should be noted that this study has limitations, such as the lack of large - scale clinical trials.

## Conclusion

This study highlights MLIP’s role in burn-induced sepsis as a potential therapeutic target. Using bioinformatics and *in vitro* analyses, we showed its regulatory effects on inflammation/cellular damage. Findings improve sepsis-cancer interplay insights, especially immune response’s role in tumor progression. Practically, they could guide targeted therapies for sepsis patients to improve outcomes. But larger-scale trials are needed for validation. Future research should clarify MLIP’s mechanistic pathways in sepsis and its oncology implications.

## Data Availability

The original contributions presented in the study are included in the article/**Supplementary Material**. Further inquiries can be directed to the corresponding authors.

## Supplementary material

Supplementary Figure 1 Analysis of promoter methylation in genes related to burns and sepsis, modulated by exercise. **(A)** ACADM Promoter Methylation: The pie chart displays methylation levels (unmethylated, low, medium, high) across samples. A bar graph shows the percentage of methylated cytosines at specific CpG sites in the ACADM promoter, and a line graph illustrates methylation patterns across various promoter regions. **(B)** ALDH4A1 promoter methylation: Similar to panel **(A)**, the pie chart, bar graph, and line graph illustrate the distribution of methylation levels, percentage of methylated cytosines, and regional methylation across the ALDH4A1 promoter. **(C)** COL12A1 Promoter Methylation: The pie chart shows methylation levels across samples, the bar graph indicates the percentage of methylated cytosines at selected CpG sites, and the line graph depicts methylation across the COL12A1 promoter. **(D)** FARSA Promoter Methylation: The pie chart illustrates overall methylation levels at the FARSA promoter, while the bar graph shows the percentage of methylated cytosines and potential methylatable sites. The line graph provides a view of regional methylation, facilitating comparison across different promoter sections. **(E)** G0S2 promoter methylation: The pie chart summarizes the methylation distribution across samples, the bar graph shows methylation percentages at various promoter locations, and the line graph details patterns across the G0S2 promoter sequence. **(F)** HSPA8 promoter methylation: Similar to prior analyses, the pie chart, bar graph, and line graph collectively display methylation levels, the percentage of methylated cytosines, and regional patterns across the HSPA8 promoter. **(G)** KCNJ2 promoter methylation: This analysis presents the distribution of methylation levels in the KCNJ2 promoter, using a pie chart for overall levels, a bar graph for CpG site methylation, and a line graph for regional analysis. **(H)** KCNQ1 promoter methylation: The pie chart, bar graph, and line diagram illustrate methylation levels, percentage of methylated cytosines, and regional methylation status across the KCNQ1 promoter segments. **(I)** LAMP2 promoter methylation: The pie chart shows methylation levels across samples, the bar graph indicates methylation at specific CpG sites, and the line chart details methylation patterns across the LAMP2 promoter. **(J)** MGME1 promoter methylation: The pie, bar, and line charts depict methylation levels, the percentage of methylated cytosines, and regional methylation across the MGME1 promoter. **(K)** MLIP promoter methylation: This section includes the distribution of methylation levels (pie chart), the percentage of methylated cytosines (bar graph), and the methylation status across promoter regions (line graph) for MLIP. **(L)** PER1 promoter methylation: A pie chart shows methylation levels, a bar graph displays methylated cytosine percentages, and a line graph illustrates regional methylation patterns for the PER1 promoter. **(M)** PHKB promoter methylation: The pie chart summarizes methylation levels across samples, while the bar graph and line graph provide details on methylated cytosine percentages and regional methylation patterns in the PHKB promoter. **(N)** PUS1 promoter methylation: A set of three charts—pie chart, bar graph, and line graph—demonstrates the methylation levels, percentage of methylated cytosines, and regional methylation across the PUS1 promoter. **(O)** RYR1 promoter methylation: This panel uses pie and bar charts to display methylation levels and proportions of methylated cytosines within the RYR1 promoter, with a line graph showing regional methylation variations. **(P)** SVIL promoter methylation: The pie chart, bar graph, and line graph visualize methylation levels, the percentage of methylated cytosines, and regional methylation status in the SVIL promoter. **(Q)** TK2 promoter methylation: The pie chart illustrates methylation levels, the bar graph shows methylated cytosine percentages, and the line graph details regional patterns within the TK2 promoter. **(R)** VAPA promoter methylation: The pie chart represents methylation levels in the VAPA promoter, with a bar graph showing methylated cytosine percentages and a line graph displaying methylation variations across different promoter regions.

Supplementary Figure 2 Analysis of exercise-influenced genes related to burns and sepsis across pan-cancer. **(A)** Mutation frequency across 20 cancer types: This panel displays the mutation frequency of genes influenced by exercise, burns, and sepsis across 20 cancer types. Each cell in the grid corresponds to the mutation frequency of a specific gene within a given cancer type. Color gradations indicate mutation percentages, allowing for a visual comparison of mutation rates for each gene across different cancer types. **(B)** Waterfall plot of mutations: This waterfall plot illustrates the mutation burden for each exercise-influenced burn and sepsis-related gene across multiple cancer types. Each bar represents the mutation load for a specific gene, with color distinctions indicating different mutation types. **(C, D)** Correlation of gene expression with Tumor Mutation Burden (TMB): These panels show a correlation analysis between gene expression and TMB across various cancers. Bubble size represents the significance of each correlation, while color denotes the direction and strength of the correlation. **(E)** Copy number amplification ratios across pan-cancer: Bars represent the proportion of samples with copy number amplifications for each gene across different cancer types, showing the prevalence of gene amplification. **(F)** Copy number deletion ratios across pan-cancer: This bar graph shows the percentage of samples with copy number deletions for each gene across cancer types, comparing deletion frequencies. **(G)** Total copy number variation (Amplification and Deletion): Each cell represents the sum of copy number changes (amplifications and deletions) for each gene across various cancers, giving a comprehensive view of overall copy number deviations. **(H)** Ratios of copy number amplifications to deletions: This panel compares amplification (positive values) and deletion (negative values) ratios for each gene across different cancer types, with cells indicating the relative prevalence of each type of copy number change. **(I)** Correlation matrix of gene expression and statistical significance (-log10 p-value) across cancer types: The correlation matrix illustrates the relationship between gene expression and p-value significance across cancer types. Bubble size indicates the p-value significance level, and color represents the direction and strength of each correlation.

Supplementary Figure 3 Pan-cancer GSVA enrichment analysis of exercise-influenced genes related to burns and sepsis. **(A)** Combined z-scores: This panel shows the combined z-scores for both normal (blue) and tumor (red) tissues across multiple cancer types. Each box plot represents the distribution of z-scores, highlighting gene set enrichment levels. P-values indicate the statistical significance of differences between normal and tumor tissues. **(B)** GSVA z-scores: This panel displays the GSVA z-scores for normal and tumor tissues across different cancer types. The GSVA method calculates enrichment scores, and box plots represent score distributions, with p-values indicating statistical significance. **(C)** PLAGE z-scores: This panel illustrates the PLAGE z-scores, which assess gene set activity levels. Box plots show the distribution of scores for normal and tumor tissues, with associated p-values to indicate significant differences. **(D)** ssGSEA z-scores: This panel presents ssGSEA z-scores, providing an additional metric for evaluating gene set enrichment. The box plots display score distributions for normal and tumor tissues, with p-values indicating the statistical significance of observed differences.
