# Supplementary figures and images for: Role of MLIP in burn-induced sepsis and insights into sepsis-associated cancer progression
Source: Front Immunol. 2025 Feb 14;16:1540998. doi: 10.3389/fimmu.2025.1540998 (PMC11868298; doi:10.3389/fimmu.2025.1540998)

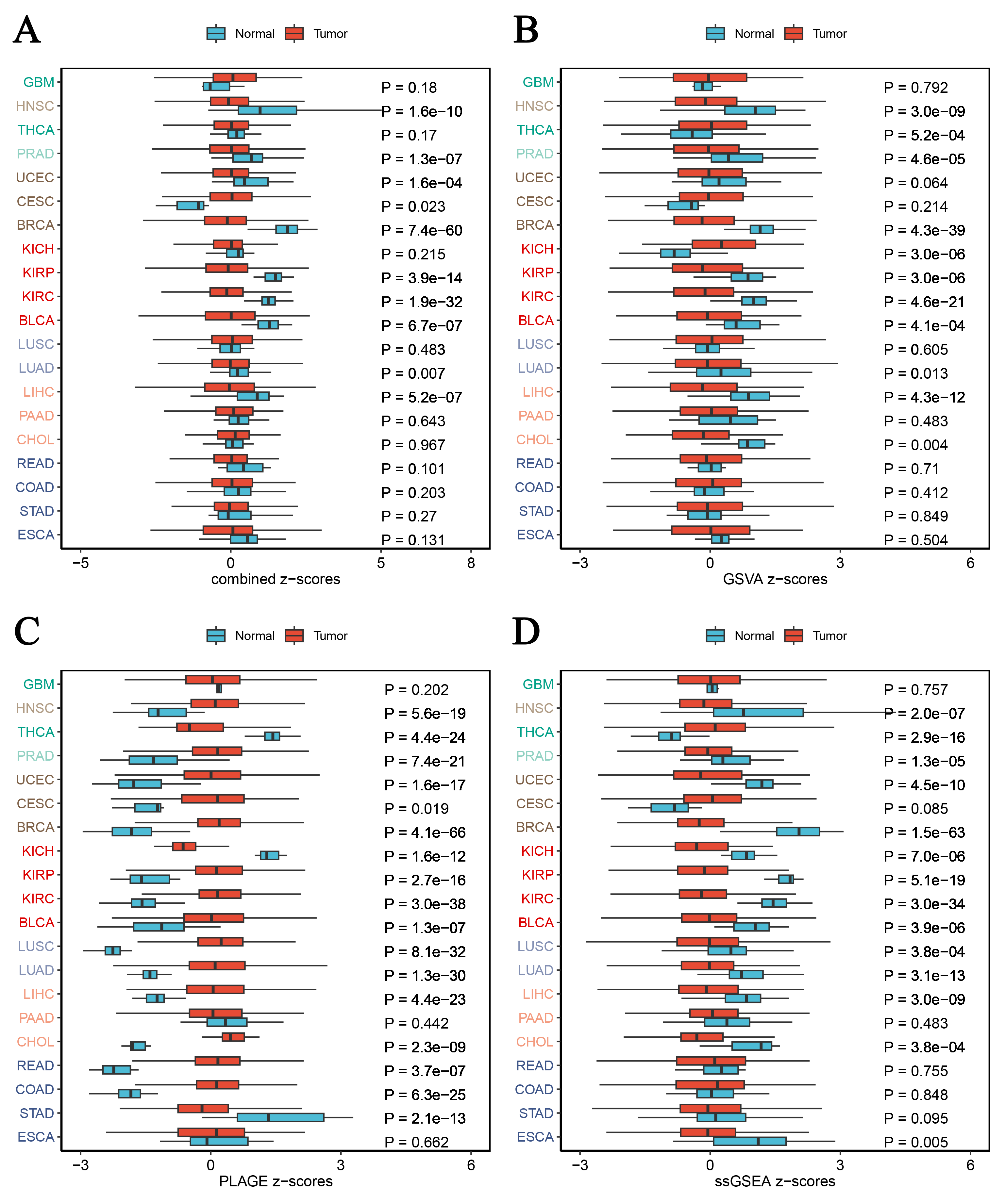

Supplement: Supplementary Figure 3 — Pan-cancer GSVA enrichment analysis of exercise-influenced genes related to burns and sepsis. (A) Combined z-scores: This panel shows the combined z-scores for both normal (blue) and tumor (red) tissues across multiple cancer types. Each box plot represents the distribution of z-scores, highlighting gene set enrichment levels. P-values indicate the statistical significance of differences between normal and tumor tissues. (B) GSVA z-scores: This panel displays the GSVA z-scores for normal and tumor tissues across different cancer types. The GSVA method calculates enrichment scores, and box plots represent score distributions, with p-values indicating statistical significance. (C) PLAGE z-scores: This panel illustrates the PLAGE z-scores, which assess gene set activity levels. Box plots show the distribution of scores for normal and tumor tissues, with associated p-values to indicate significant differences. (D) ssGSEA z-scores: This panel presents ssGSEA z-scores, providing an additional metric for evaluating gene set enrichment. The box plots display score distributions for normal and tumor tissues, with p-values indicating the statistical significance of observed differences. [file Image3.png]
